# Carp Breeding in the Carpathian Basin with a Sustainable Utilization of Renewable Natural Resources

**DOI:** 10.3390/life12101661

**Published:** 2022-10-20

**Authors:** Laszló Horváth, Éva Kovács, Balázs Csorbai, Árpád Hegyi, Kinga Lefler, Tamás Müller, Béla Urbányi

**Affiliations:** 1 Institute of Aquaculture and Environmental Safety, Hungarian University of Agriculture and Life Sciences (MATE), 2100 Godollo, Hungary; 2 Independent International Consultant, 1063 Budapest, Hungary

**Keywords:** common carp, carbon footprint, carp farming method, environmental protection, energy transfer, water ecosystem

## Abstract

In the Central European region, there is a long tradition of breeding fish in artificially constructed ponds. As the area belongs to the temperate zone, farmed fish need to survive cold winter periods. Common carp (*Cyprinus carpio* L.), which is an omnivorous, bioturbating species, is well adapted to warm and cold periods and the alluvial water environment. Since the Middle Ages, a large scale, efficient carp farming methodology has been developed in the region, where production is based on natural resources (protein and fatty acid sources) of renewable water ecosystems. This summary aims to present this well-developed breedi:ng method through discussing aspects of hydrobiology and energy transfer through the food chain as well. Capabilities and effects of agro-technical treatments such as liming and organic manuring, zooplankton management and possible supplementary feedings are also reviewed. Analysing chemical processes of waters uncovers that biological production has no carbon footprint; no carbon dioxide is released into the atmosphere. In contrast, gaseous carbon dioxide diffuses into pond water containing calcium and/or magnesium, then it accumulates in algae production and, through energy migration to upper trophic levels, increases carp production. Thus, it can be declared that pond-farmed carp provides an environmentally friendly, delicious meat among products of animal origin.

## 1. Introduction

### 1.1. Development of Carp Breeding Method

In the prehistoric ages, besides other fishes, our ancestors hunted for large carps, which spawned in shallow areas of lakes and rivers. In the period of agriculture development, early farmers started to protect small water bodies with fish in them, staying behind from river floods. People used these little basins as food reservoirs and started to nurse fish in them. Later, artificially made small basins were used for keeping fish alive for longer periods of time, so-called “piscina”, during the age of the Roman Empire. Nowadays, common carp (*Cyprinus carpio* L.) is considered to be the first and only domesticated fish species, which is farmed for human consumption [1,2].

In the history of carp breeding and domestication, the earliest written publication of a successful carp breeding originates from a Chinese author, from the 5th century BC. Fish farmer Fan Li provided a detailed description of the conditions necessary for the successful breeding of carp [3].

He emphasized the importance of small heaps in the spawning pond with the sides covered by dense grasses, to provide excellent spawning sites for the sticky eggs of carp.

In Europe, a Czech bishop, Dubravius, who lived in a monastery, farmed carp in the Middle Ages, during the 16th century. In his famous book written in Latin, he had statements about reproduction and feeding that are still valid today [4].

In the 19th century, the Silesian fish farmer Tomas Dubits developed a complete carp breeding method, which produced the most efficient seed stocks for fishponds for many decades. His method was mainly applied in Central Europe. In Hungary, the famous polyhistor Otto Herman disseminated this efficient method of carp propagation [5].

In his excellent book (*A short description of Fish Farming*, 1888), Herman gives a detailed information about the Dubits method. According to the author, who lived near to the river Danube, Dubits observed which environmental factors led to/motivated the spawning of mature wild carp and reproduced these factors within farm conditions. During late spring, when the water temperature reached 18 °C, he used small, shallow, man-made ponds covered with short grass to propagate carp. Mature carps were transported into the newly inundated ponds, where reproduction started within 2–3 days, as it happens on freshly flooded areas of natural rivers.

In nature, spawning carps stick their eggs to grasses where the developing carp eggs have access to oxygen dissolved in the water during embryogenesis. So, the sticky surface of freshly ovulated carp eggs is an advantageous adaptation technique, as without this, eggs would fall down into the mud and die in the anaerobic sediment.

After hatching, non-feeding carp larvae hang on the grasses up to 3–5 days. They start to feed when they fill up their swim bladder with air. In the Dubits method, carp larvae live in the small ponds up to 10–14 days. In this period, the name of the juveniles is “mosquito” carp, as their size is similar to that of mosquito larvae. At the end of this short period, young carps are collected with fine nets and transported into nursing ponds.

At the end of the 19th century, many carp rearing ponds were established around the Central and Eastern Europe for carp breeding. World War I broke this process of development, and only years after the war was a progressive growth regained. Between the two World Wars, intensive research started on the field of limnology (freshwater hydrobiology) in Central Europe. These studies helped to understand the biological production of carp breeding ponds. Some famous scientists (such as Schaperclaus [6] and Maucha et al. [7]) recognized that the lowland-type areas—including the Carpathian Basin—have many advantages specifically for carp breeding. Based on their suggestions, new fish breeding units were established intensively. Limnologists determined optimal levels for several environmental factors limiting biological production (e.g., algal development). According to their results, inorganic carbon compounds (carbonate/hydrocarbonate anions) together with calcium and magnesium ions provide a stable, buffered water environment/status and a continuous source of carbon for one-cell algae production. The optimal level of these substantial chemical compounds of water is fundamental for a high biological production in shallow ponds. The role of other critical factors such as light, level of other nutrients (such as N and P), as well as the trophogenic layer in the upper area of ponds was also investigated and optimal levels were determined. On the basis of the ideas of Maucha, Woynarovich [8], a new and very effective “carbon manure” method was elaborated. By using frequent, daily introduced pig manure, the biological production of the pond—most importantly the biomass of zooplankton—was increased. With this method, the amount of market-size carp per hectare could be doubled (1500–2000 kg ha^−1^ of production could be reached in a large fish farm, where the pond area was more than 300 ha). For comparison, at that time the average carp production of Hungary was 500–600 kg ha^−1^ [8].

Following World War II, the development of the fishery sector was renewed/restarted. To be able to adapt to previous research results, the need for young carps expanded for stocking the ponds.

To satisfy the enlarged demands for young carp, contemporary scientists and farmers searched for more effective propagation methods to utilize the huge potency in carp reproduction. It was already well known that in general the most efficient techniques for incubating fish eggs were vertical incubators such as Zug jars, mainly used for Salmonids and Coregonids. While the stickiness of carp eggs is very useful for natural spawning, as it prevents the eggs from falling into the mud, it causes huge problems within hatchery conditions. In fresh water, newly fertilized carp eggs stick together within seconds after being placed into Zug jars for incubation. However, due to the great demand, the elimination of the stickiness of carp eggs became a main aim for scientists.

To remove adhesive proteins from the egg surface, intensive international research work started in the early 1960s. Ristic and Jovanic (1960) used starch solution [9], Tec (1963) [10] and Konradt and Szacharov (1963) applied hyaluronidase enzyme [11], while later on Magomajev (1976) treated carp eggs with diluted cow milk [12]. Woynarovich (1962) adapted a different, simple idea: he used a mixture of common salt and carbamide solution [13]. This simple and popular method dominates in carp hatcheries even today as it is efficient, simple to use and does not contain any organic compound.

For efficient carp breeding, not only is a successful egg treatment required, but also the programming of the ovulation of mature female carp. Von Ihering (1937) from Brasilia published results on a full ovulation of teleosts. He treated endemic ornamental fish with pituitary extract and sustained complete ovulation with fertile gametes [14]. Large-scale hypophysation and induced ovulation in an endangered group of fish, the acipenserids, was elaborated by Gerbilskij and his co-workers in 1939. This was the first adaptation of hormonal induction which had a commercial value [15].

In countries where carp-based foods were popular, for example, in Central-Eastern Europe, carp hypophysation started during the early 1950s. Within a few years, a large-scale method for hatchery production was developed [16,17].

In the early years, this explicit hatchery method resulted in the production of millions of carp fries; however, their survival rate was very modest [18]. Observations on the area of successional/consecutive changes of zooplankton in freshly inundated nursing ponds resulted in a vital improvement. The main problem was that harmful predatory zooplankton groups (copepods) killed a large number of young carp stocked into nursing ponds. Tamas & Horvath recognized the cause of the low survival of fish larvae and suggested a pond preparation process together with a zooplankton selection which resulted in better water conditions by the time young carps were put into the pond [19]. As a result, the survival rates of carp fries significantly increased [20].

### 1.2. Characterization of Carp Breeding in the Region of Central Europe: Advantages and Disadvantages

In the Carpathian basin and its surrounding areas, carp production has a very special pond breeding technology, which requires some basic environmental conditions for success as follows:

– A large land-type area to establish shallow ponds, as the optimal production volume of marketable carp is only 700–1500 kg/hectare (under temperate climate). Thus, a large pond surface is necessary to be feasible.
– The water of freshwater ponds should have a high Ca/Mg content to ensure stable and buffered pH conditions for a daily production of algae.
– Cheap sources of organic manure (cow, pig, poultry, etc.) or plant-based organic biomass should be available to provide optimal levels of biogenic elements for biological (single-cell algae) production. It is important to remark that organic manure transferred into the pond secures different elements of nutrients, including carbon compounds for biological production, and is not a direct food for fish!
– Large amounts of grain or agricultural by-products produced in the surrounding agricultural areas can significantly reduce production costs.
– A production season longer than 100–120 days (with an average temperature higher than 10 °C).
– Steadily attainable markets which prefer products of fresh or processed carp.

If these conditions prevail, very good quality carp can be produced by using this method, with high economic profit for many decades. It is well known that many artificially established ponds have been producing carps for hundreds of years.

At the same time, this carp breeding method utilizes renewable natural resources of water ecosystems, such as planktonic and benthic communities of fresh waters.

The Carpathian basin has good carp production facilities: the region has large fields, where shallow carp breeding ponds can be established.

Water comes from the surrounding Carpathian Mountains, which is rich in calcium-magnesium minerals originating from the sedentary shells of dead marine creatures from the former Pannonian Sea. Waters from surrounding mountains run down to land areas. Rivers with high calcium and magnesium hydrocarbon content from limestone-type mountains and hills transport water to fishponds.

Countries in the Carpathian basin and surrounding areas have well-developed breeding technologies and remarkable stocks of different domesticated animals; therefore, sources of organic manure may normally be available for pond treatments.

A huge part of the human population traditionally follows Christianity and prefers fish dishes (mainly of carp origin) during important holidays such as Christmas and Easter.

Other benefits of this fish production method are that the end products (marketable carp) are delicious and healthy due to the high unsaturated fatty acid and digestible protein content. No heavy metals are present in the fish flesh, as there are no mines of heavy metals in the mountains surrounding the water catchment area. From a physiological point of view, the flesh of freshwater fish is safe compared to that of marine fish, which may contain different toxic heavy metals.

From an ecological perspective, this breeding method utilizes the evolutionary adapted zooplankton and zoobenthos as sources of protein and essential fatty acids for fish, which is developed naturally in pond ecosystems.

Later, water chemical processes will be analysed more deeply, and it will be proven that carbon dioxide, which circulates in the carbon-cycle of water, is not able to leave it. Consequently, biological production based on the algae—zooplankton and zoobenthos food chain has no carbonic footprint. Only supplementary, grain-based feeds and daily technical activities have limited footprints; therefore, carp production is one of the best environmentally safe animal breeding technologies.

### 1.3. Why Carp? Special Feeding Habits and Ethological Properties of Omnivorous Carp

Among bony fishes (Teleosts), common carp (*Cyprinus carpio*) is a well-adapted, advanced fish species. During the evolution of fish, carp as an independent species evolved in areas of Central Asia after the last glacial period. Many groups of basic subspecies (*C. c. aralensis*) emigrated from there, both in eastern and western directions. Over the centuries, the basic form developed into different subspecies. During migration, carp from the Aral region reached Amur River (eastern variety *C.c. haematopterus*) and the water system of Danube through the brackish, shallow area of the Black Sea (western type carp *C. c. carpio*).

Ancient carp was a very adaptive species, it was able to acclimatize to prevailing feeding sources; therefore, it became an omnivorous fish, which could consume food not only of animal origin, but of plant origin as well, e.g., different seeds, roots and sprouts of water weeds and swamp vegetation. Thanks to this important characteristic, carp was able to occupy different habitats of shallow lakes and rivers in temperate and subtropical areas [21].

**Figure 1:**
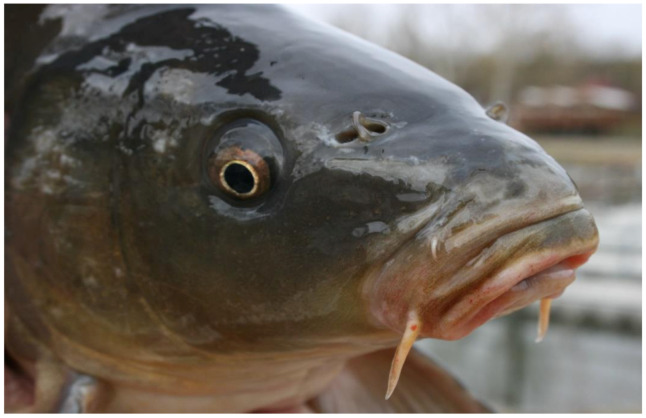
Closed mouth of carp with a moustache, which helps to find food organisms in the mud (Photo: A. Hegyi).

**Figure 2:**
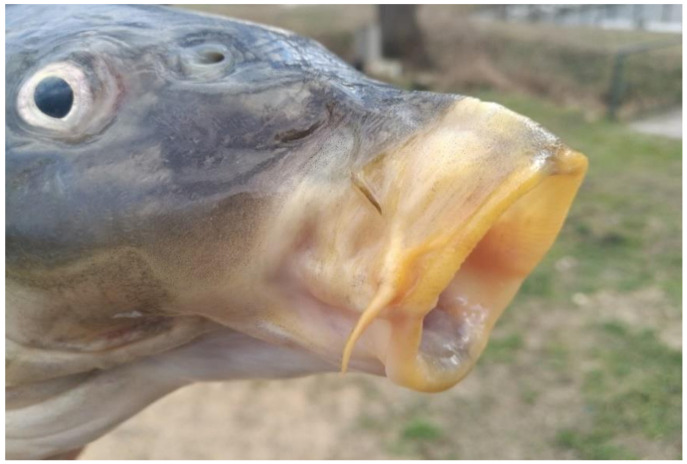
Extended mouth of carp suitable for bioturbation (Photo: A. Hegyi).

Due to the omnivorous feeding strategy, the mouth of the carp is multifunctional (see Figure 1 and Figure 2).

During the feeding process of carp, zooplankton is filtered from the open water of ponds or from invertebrates such as worms, small mussels and snails from the mud.

The lingual bone in the mouth of the carp and the flexible Weberian apparatus connecting the internal ear with the fish bladder play a major role in the selection of food. Food organisms selected in the mouth shift towards the pharynx. In the case of hard food particles, e.g., grain seeds, the strong pharyngeal teeth break them down.

When a carp searches for food among organisms living in the lake sediment, it causes significant changes for the pond ecosystem due to its feeding habit, as it absorbs a large amount of lake sediment, like a vacuum cleaner. After selecting food particles, the rest is blown/moved back (organic sediment, clay colloids, etc.) into open water. In this oxygen-rich medium, aerobic bacteria adhere to organic particles and immediately start to decompose them.

Bacterial and micro-fungal degradation of dissolved or particulate organic matter produces carbon dioxide, which instantly reacts with non-soluble microcrystals of calcium and magnesium carbonate to form water-soluble calcium or magnesium bicarbonate. During their assimilation, microscopic aquatic plants, such as algae and cyanobacteria, take up carbon dioxide from this bicarbonate compound and produce simple sugars and organic macromolecules, while increasing the biomass of algae. Thus, the decomposition of dead organic matter simultaneously creates favourable conditions for the construction of new, energy-rich organic matter. Consequently, carp feeding in the sediment causes a resuspension of non-living organic matter, which results in a circulation of organic matter in the aquatic carbon cycle.

Knowledge and ability to manage the carbon cycle of fishpond ecosystems is essential for the economic operation of a pond fish farm. All recently implemented agrotechnical interventions to increase fish production are closely related to the carbon turnover of lakes and the utilization of renewable natural resources.

Therefore, the present review of the concept and practice of organic fish production in the Carpathian Basin starts with an overview regarding the basis of the following supporting sciences, hydrobiology and ecology.

## 2. Biological and Hydrobiological Background of Pond Carp Breeding

The tiny outer surface of our planet is called the geographic envelope. All the physical, chemical and biological events happen within this envelope, which is divided into different spheres.

A characteristic part of this envelope is the gaseous atmosphere, which covers the whole surface of our planet. This surface is broken up to form continents, islands, icebergs, glaciers, water bodies (oceans, seas) and inland waters within land boundaries. A few billion years ago, organic life on Earth was born in shallow, warm marine water. Nowadays, the majority of living organisms still exist in water environments. An important part of the surface is called the hydrosphere, which includes all types of waters and is inhabited by aquatic organisms.

The lithosphere is formed by soils and rocks of lands, and it also includes the pedosphere, which is the upper 8 m layer suitable for terrestrial life, such as current human societies. Some living organisms require both terrestrial and aquatic environments, while thousands or millions of terrestrial species live in the pedosphere and in lower layers of the atmosphere.

Following the appearance of organic life, ancient living organisms inhabited all mentioned spheres, establishing the biosphere. While they occupied terrestrial and aquatic areas, they also modified their inorganic, physical and chemical environment. A good example of such modification is the activity of ancient aquatic water plants (algae and cyanobacteria), which created an oxygen-rich atmosphere for Earth over millions of years. A similar event is the current global warming aggravated by human activities through the large-scale utilization of solid and liquid carbon deposits.

In the following, a fish farming technology operating in a non-terrestrial, aqueous environment will be analysed. Experts working in aquatic environments need some basic information on the chemical and biological processes of fresh waters, mainly pond water, to be successful in fish production. The science of hydrobiology helps them to understand and regulate processes supporting fish production. Before discussing fish breeding technologies, some important facts, definitions and roles of hydrobiology are considered and discussed.

Subject to the ionic level, water bodies of the hydrosphere can be divided into two large units: (a) seas, where the ion level is high and relatively stable, and (b) fresh waters with low and variable ion levels.

Oceans, seas and inland seas resulting from marine transgressions belong to category A. Generally, a high salt content (3.5%), mainly sodium chloride, characterizes these waters. The higher the dissolved ion content of the water is, the higher its density is.

The status of waters in category B is more variable. For certain lakes that exist on special minerals/soils, the water can contain very high ion levels (soda lakes).

Other extreme cases are waters of rainy tropical areas which are nearly distilled. In general, inland waters have low ion levels.

Categorization of inland waters based on their total ion content results in three main groups:

1. Fresh waters: with a total salt content of 0–500 mg/L, with 0–700 µS/cm specific electrical conductivity.
2. Transitional waters: 500–3000 mg/L salt content.
3. Salt waters: above 3000 mg/L salt content.

The salinity of seawater is 35,000 mg L^−1^, approximately 3.5%, with a specific conductivity of around 70,000 µS/cm.

Waters can also be categorized according to the movement of water (lakes and rivers), depth (deep or shallow), origin (natural or artificial), length of existence (long life or astatic), etc.

### 2.1. Hydrological Cycle

**Figure 3:**
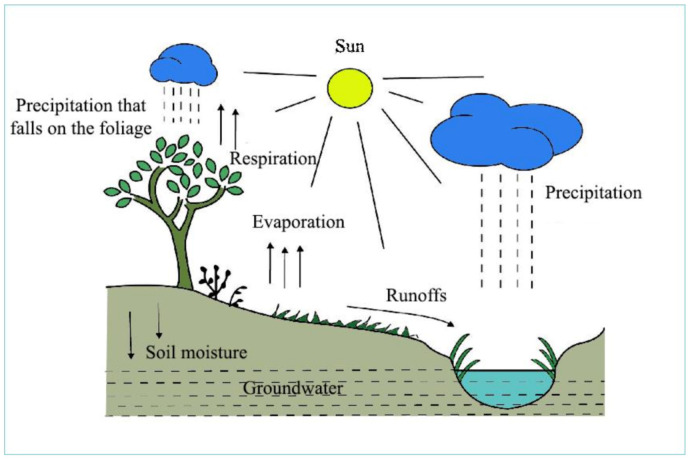
The hydrological cycle, water circulation of the geosphere (Padisák, 2005; modified) [22].

Water molecules are moving constantly. During evaporation and plant transpiration, water molecules emerge from surfaces of terrestrial areas and water bodies (oceans, lakes, rivers etc.), thus creating clouds. At a certain moisture content, after cooling, rain and snow develop in clouds, fall as precipitation and the circulation starts again (Figure 3).

Water management has a significant importance for human populations, as freshwater is becoming a limited renewable resource. Water is needed both for humans and agriculture, including animal breeding, plant production and a sustainable natural life. Nowadays, the human population is gradually increasing and needs more water, while potential fresh water sources remain the same or are even decreasing. Escalated by global warming, the role of water will be more significant in the future.

### 2.2. Physical and Chemical Properties of Water Molecules

Water is essential for life. Every living creature, from the simplest microorganisms to the most advanced ones, needs water.

H_2_O is the most common molecule in the world and is the only compound that is present in three forms at the same time: gas, liquid and stable (microcrystalline) phases.

**Figure 4:**
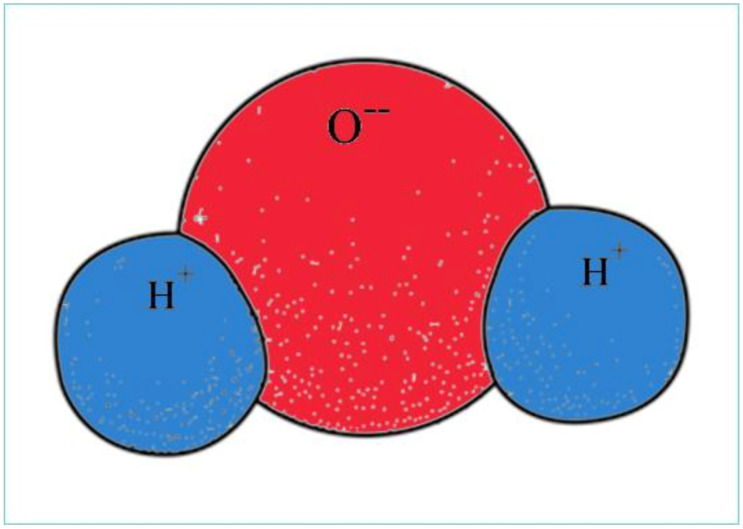
Bipolar water molecule.

The structure of a water molecule explains all special properties that are characteristic for the aquatic environment. In the water molecule, two hydrogen (H) atoms with lower electro negativity are connected to a single oxygen (O) atom with high electro negativity by a strong covalent bond. The three atoms are not arranged along a linear axis, but the oxygen and the two hydrogens are positioned at an angle of 105 degrees, thus creating a dipolar molecule (see Figure 4).

Unbound negativity of the negatively charged oxygen also attracts the two hydrogen atoms of other water molecules. However, this bond is a weaker, secondary chemical bond, called a hydrogen bond. Accordingly, water molecules form smaller clusters, establishing a crystal-like structure.

Oxygen atoms with a slight negative charge and hydrogen atoms with a slightly positive one within a water molecule not only attract adjacent water molecules but are also able to connect to other charged atoms and compounds. Due to its bipolarity, water makes for an excellent solvent. The connection of water molecules with other atoms and ions is mutual, because atoms and compounds with different charges are formed from compounds dissolved in water, which can have a negative or positive charge. Bipolar water molecules form a shell around ions. The process is called electrolytic dissociation. On the other hand, water molecules can also form a hydrated shell around water-insoluble organic macromolecules (e.g., fats); thus, spherical droplets are developed.

The dipolar nature of water molecules also explains the specific density–temperature relationship of water (temperature anomaly).

**Figure 5:**
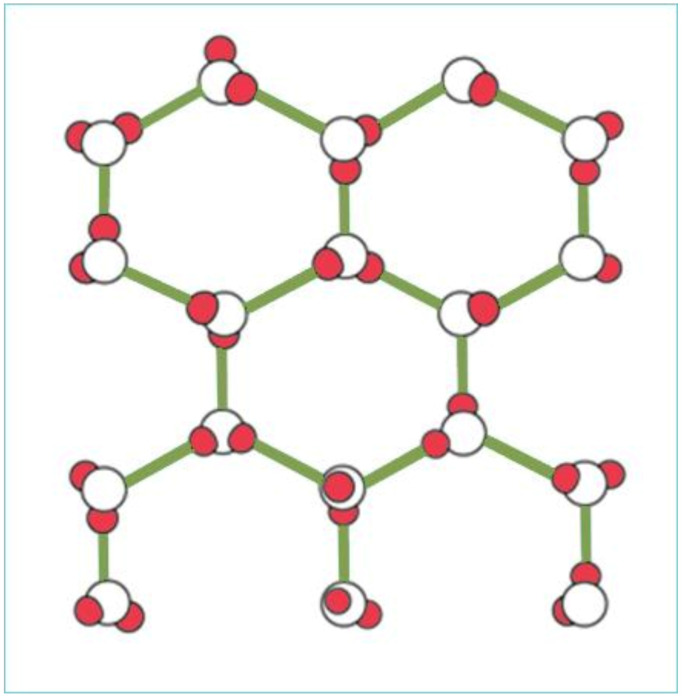
Crystal structure of water molecules in ice.

In its solid phase, as ice, water molecules are arranged in a crystal lattice and are fixed (see Figure 5), where the distance between the atoms is constant. The volume of solid ice increases when compared to liquid water, due to the crystal lattice. Its specific gravity is less than that of water; as a result, ice floats on liquid water. During the melting of ice, the arrangement within the crystal lattice gradually disappears. The distance between molecules/atoms is largest at freezing point.

Additionally, there is another physical effect that affects the density of water: it increases the distance between the molecules/atoms due to heat transfer and is called Brownian motion.

The two processes influencing water density have antagonistic effects. Crystal lattice formation of gradually cooling water does not yet start at +4 °C, so there is no increase in the volume. Contrarily, thermal expansion at +4 °C is the lowest; thus, the specific gravity of water is the highest. This particular property is exceptionally important for aquatic life and is ultimately the result of the molecular structure of water.

So, the maximum density of water is above its freezing point, exactly at +4 °C. This phenomenon is called the temperature anomaly of water, which helps the deepest layers of water bodies to stay constantly at +4 °C, saving living organisms from freezing.

Water density is also affected by salinity (ion concentration), where the relationship is straight (linear). The higher the dissolved ion content of water is, the higher its density is (seawater).

### 2.3. Heat Capacity and the Route of Sunlight in Water

A further important physical property of water is its ability to absorb heat. Water warms up and cools down slowly when influenced by a changing temperature, as it has a very high heat capacity and can store a large amount of heat.

Thermal stratification of stagnant waters is related to the maximum density at 4 °C. In spring, the upper layer of waters begins to warm. When the temperature of lake water reaches 4 °C, the temperature of the whole water body becomes identical due to turbulence and vertical currents, and the temperatures of the upper epilimnion and the lower hypolimnion are equalized. In summer, the lighter warm water is located in the upper layer, forming again an epilimnion and a hypolimnion. In autumn, the process is reversed and allows for mixing to occur.

Due to the effect of wind and the movement of large fish stocks, only short-term thermal stratification occurs in shallow fishponds.

Sunlight has prominent physical and physiological impacts in water. When the biosphere is considered, this fundamental and decisive radiation is made up of mutagenic ultraviolet, visible and infrared ranges. The light absorbing ability of water is an important physical feature. In water, the light with different wavelengths is absorbed in different ways. Visual and infrared ranges are required for plant (algae) assimilation. In clean and transparent waters, the long waves of blue light from visible light penetrate the deepest [22,23].

Sunlight can only penetrate 20–30 cm deep into turbid water, while some of its heat is absorbed in the upper water layers. These layers are the best breeding mediums for unicellular algae; thus, experts of pond aquaculture should maintain the water turbidity of lakes. Bioturbation of carp and artificial agitation of the sediment can obscure pond water.

### 2.4. Cycles of Biogenic Elements within Aquatic Conditions

In addition to liquid water, inorganic carbon compounds (carbon dioxide CO_2_, hydrogen carbonate anion HCO_3_ and carbonate anion CO_3_) and biogenic elements, such as nitrogen and phosphorous, need to be present in pond water to enable organic production. Certain cations that bond with carbon compounds, especially magnesium and calcium cations in the aqueous medium also play a key role in pond production (further discussed in the next chapter).

From an environmental point of view, it is particularly important to analyse chemical bonds of elements at an atomic level in order to prove that carbon dioxide that has entered or was generated in pond water cannot escape into the atmosphere; it does not increase the level of carbon dioxide in the air but circulates in the system until being fixed by algae.

For a better understanding and management of the biological production and energy transfer of pond ecosystems, basic structures and reactions of elements/compounds at the atomic level should also be considered. These basic structures and reactions can be investigated by using Bohr’s atomic model and can explain biological processes in ponds.

**Figure 6:**
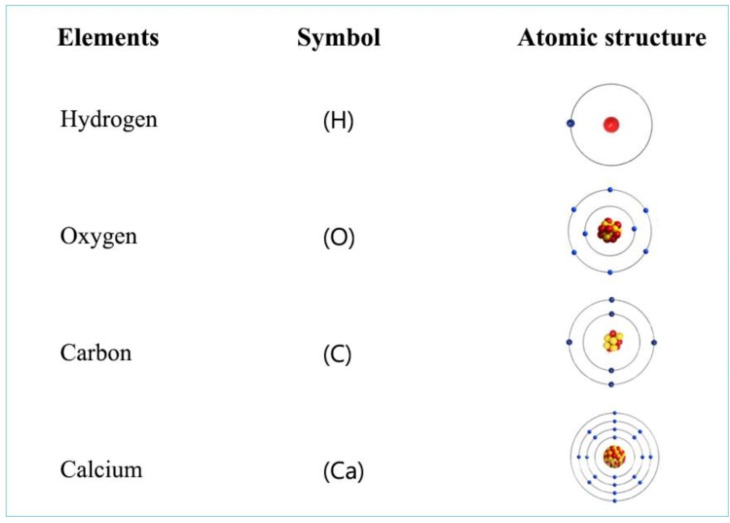
Atomic structures of a few biologically important elements.

Atoms of various elements occurring on our planet are made up of an atomic nucleus and electron shells around it (Figure 6). The attraction between these two components ensures the stability of atoms. The nucleus is made up of protons and neutrons. In the electron shells, electrons are located around the nucleus, the quantity of which depends on the number of protons in the nucleus. These electrons consist of pairs. When combining elements, compounds are formed, the stability of which is established by their common pairs of electrons.

Larger elements with more protons and thus more electrons have more subshells within the electron shells. The electrons on the outer layer are the so-called binding electrons. They also try to form electron pairs and aim for a filled subshell. For that reason, missing electrons are taken from or excess electrons are transferred to other elements. The origin of the electron pairs formed in the binding electron shell determines the type of chemical bond between different elements (covalent or ionic bond, etc.) and the charge of the elements and compounds. Negative ones with an electron excess relative to the proton number are called anions, while positively charged ones with an electron deficiency relative to the proton number are called cations.

**Figure 7:**
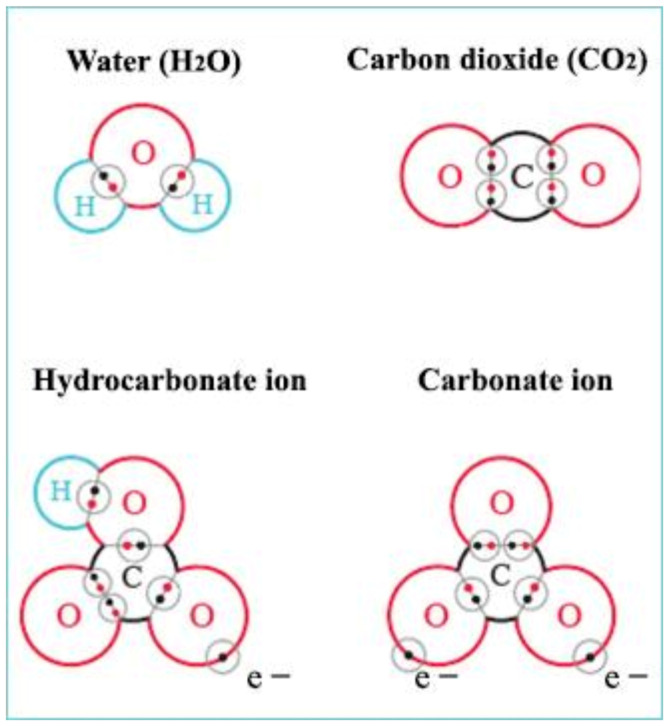
Binding types of certain compounds important in the biological production of ponds. Water molecules and gaseous carbon dioxide have purely covalent bonds, while hydrocarbonate and carbonate anions have both covalent and ionic ones.

Four electrons are located in the outermost electron shell (called valence shell) of a carbon atom. As it would require eight electrons for saturation, four more are needed. How can the carbon atom reach this status? The simplest stable compound of a carbon atom is gaseous carbon dioxide, the structure of the outer binding electron shell of which is shown in Figure 7. Missing electrons are received from two oxygen atoms and the type of the chemical binding is covalent, which is a very stable connection among atomic bindings.

**Figure 8:**
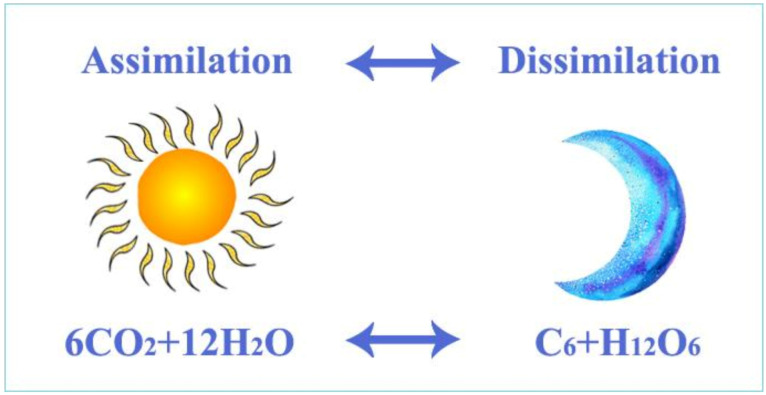
A basic equation of the assimilation of water plants, mainly algae. During the process of assimilation, algae synthesize high-energy organic molecules from carbon dioxide molecules by using the energy of sunlight.

Algal assimilation generates proper conditions for the production of organic matter and energy in lakes (Figure 8). The process requires carbon dioxide, water molecules, sunlight and functional chlorophyll of algae. During the daytime, algae synthesize organic matter (sugar and organic macromolecules such as starch, protein, fat, vitamins, hormones, etc.) that are incorporated into the algal biomass. Through the aquatic food chain, algae-consumers transform a small portion of this organic matter into their body material; the energy produced by the algae moves to higher trophic levels. Biocenosis (all living organisms in a pond) uses some of the energy produced during the day for metabolism and produces carbon dioxide during dissimilation. When the result of assimilation is investigated, it is recognised that the difference between production and decomposition is the net biological product.

In an aquatic environment, depending on the pH of water, only a very small proportion of carbon dioxide can be directly absorbed by algae. At a neutral pH, most of the inorganic carbon is present as hydrocarbon or carbonate anions. Before discussing the circulation of all carbon compounds in aqueous environments, it is necessary to define what pH is: pH and pOH specify the acidity or alkalinity of water (the presence of free hydrogen or hydroxyl ions).

**Figure 9:**
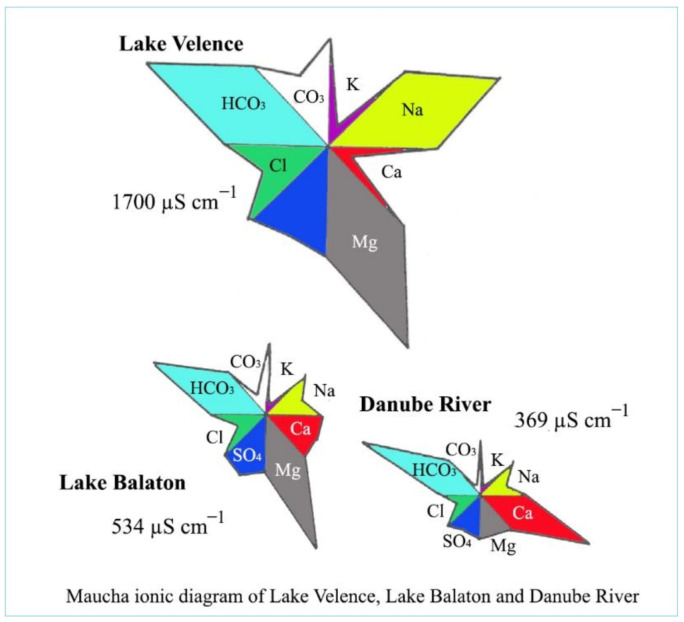
Maucha diagrams of some waters in the Carpathian basin.

Fresh waters not only contain carbon compounds and calcium and magnesium ions, but also many other elements which influence the total ionic character. Clean water can be characterized by a high solubility. From minerals and soils, different ions can be dissolved into water. For a chemical characterization of water, four main cations and anions need to be determined: these cations are Ca^2+^, Mg^2+^, Na^+^ and K^+^, and the anions are HCO_3_^−^, CO_3_^2−^, Cl^−^ and SO_4_^2−^. To demonstrate these ions quantitatively in a water body, the Maucha diagram (Figure 9) can be used. Separate peaks of different ions indicate their amounts as well.

The final value of water pH evolves from the overall amount and type of single ions in the water.

As is presented in Figure 9, the waters of the Carpathian basin are characterized by calcium/magnesium hydrocarbonate, which is the result of the ion content of soils and minerals of the surrounding hills. The pH value of calcium and magnesium hydrocarbon types of waters is around 7–8, which belongs to the neutral category. Not only is the neutrality of these waters beneficial, but so is their pH stability, as living organisms prefer a stable pH for their metabolic processes.

**Figure 10:**
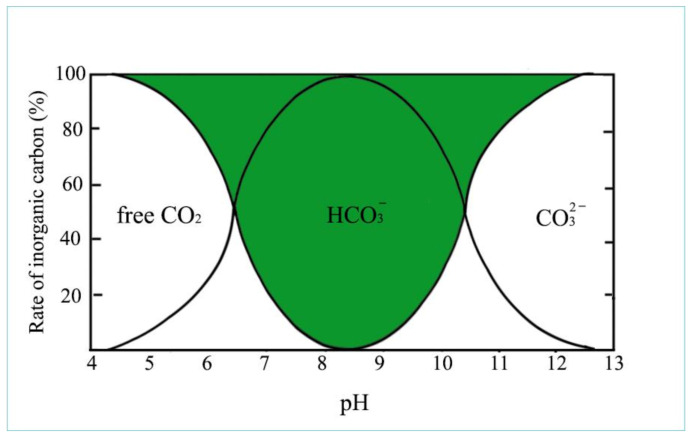
Forms of inorganic carbon compounds based on pH level (Padisák, 2005, modified).

**Figure 11:**
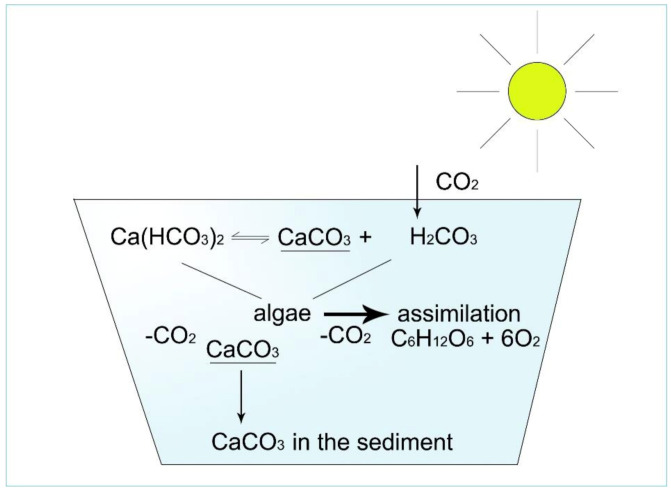
Well-buffered pond water contains calcium hydrogencarbonate as a carbon dioxide reservoir for algae. As a result of assimilation, daytime CaCO_3_ crystals are produced; thus, water pH remains stable.

**Figure 12:**
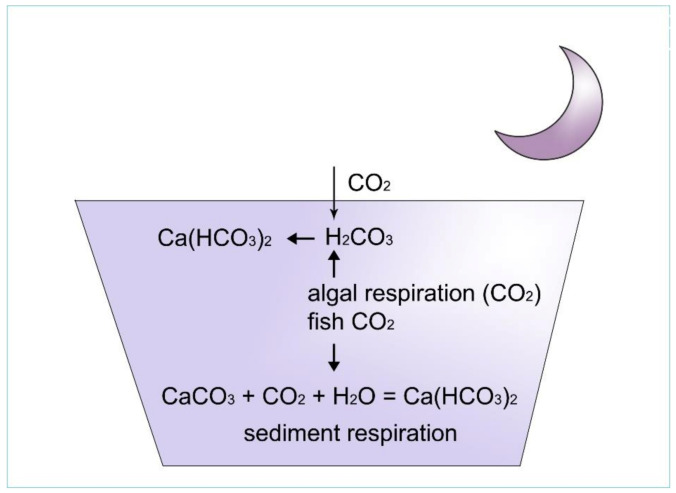
At night, free carbon dioxide reacts with calcium carbonate crystals and water to produce calcium hydrogen carbonate. Thus, the pH of water remains steady.

**Figure 13:**
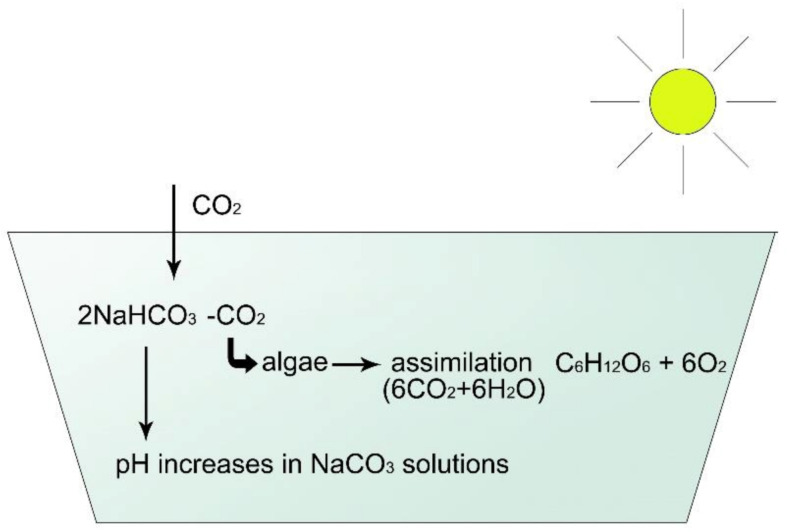
In sodic ponds, pH increases during the daytime because of assimilation because sodic Na_2_CO_3_ remains dissolved.

**Figure 14:**
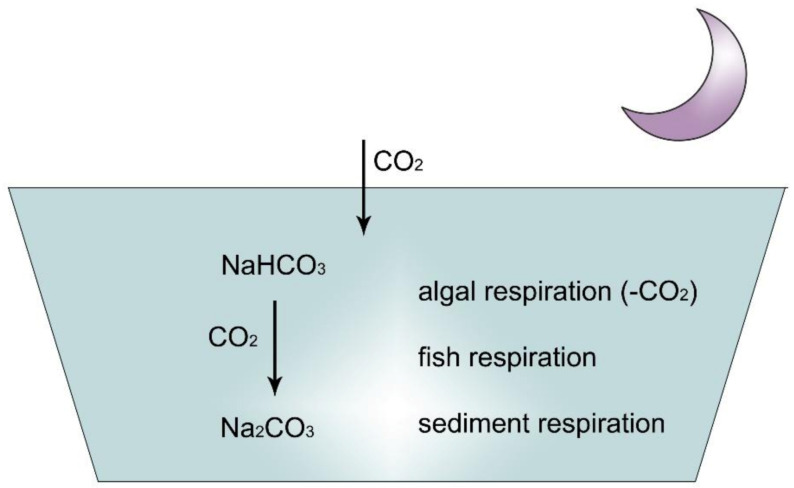
At night, due to carbon dioxide, pH decreases in sodic ponds.

How can pH be stable in these types of water? To understand this phenomenon, the effect of algal assimilation on inorganic carbon circulation must be analysed (Figure 10, Figure 11, Figure 12, Figure 13 and Figure 14).

### 2.5. Inorganic Carbon Circulation of Aquatic Ecosystems

Figure 10 provides an overview of the appearance of inorganic carbon compounds that depend on the pH of water.

As illustrated in Figure 10, in acidic conditions (pH 4–6), all inorganic carbon occurs as dissolved free CO_2_, which create carbonic acid (H_2_CO_3_) with H ions, while at alkalic level (pH 11–14), carbonate anions dominate. In the neutral pH range, 100% of carbon is present as bicarbonate (green field), mainly in the form of hydrocarbon anions and about 1% as so-called non-equilibrium carbon dioxide. This is the form of CO_2_ which can be used up by algae through osmotrophy. When algae consume this small amount of CO_2_, new hydrocarbon molecules dissociate into CO_2_ and carbonate ion for maintaining the equilibrium, as is described in the following:

Anions are connected to different cations, forming an ionic bond compound. The strength of electronegativity of cations decreases as follows: Ca, Mg, Na, K. Therefore, HCO_3_ anions will join first to Ca cations. During the assimilation process, algae will first ingest free equilibrium CO_2_ from Ca(HCO_3_)_2_ as:

Ca(HCO_3_)_2_ = CaCO_3_ + H_2_CO_3_

H_2_CO_3_ = H_2_O + CO_2_

Fortunately, the end product of this process is CaCO_3_, a crystalline compound (calcite) which immediately descends into the sediment, and the pH of water remains neutral. The process is the same with magnesium, resulting in MgCO_3_ (called crystalline dolomite). This is why a stable pH characterizes well-buffered Ca/Mg type of water.

In contrast, when no Ca or Mg cations are present but Na^+^ or K^+^ are available, the pH does not remain stable:

2Na(HCO_3_) = Na_2_CO_3_ + H_2_O + CO_2_

Na_2_CO_3_ is not a crystalline compound and remains in the solution as soda, which increases alkalinity! During the daytime, when assimilation is intensive, pH in sodic water can increase to high, toxic levels.

The high level of Ca and/or Mg in waters of the Carpathian Basin results in stability and high production efficacy of water vegetation, including algae [24]. 

**Figure 15:**
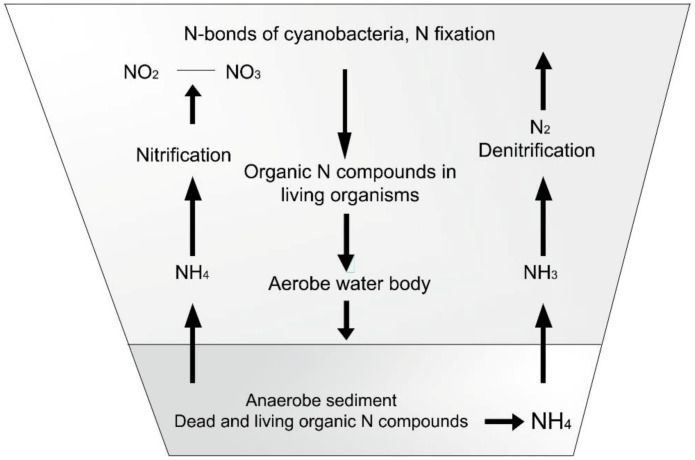
Nitrogen cycle in ponds.

**Figure 16:**
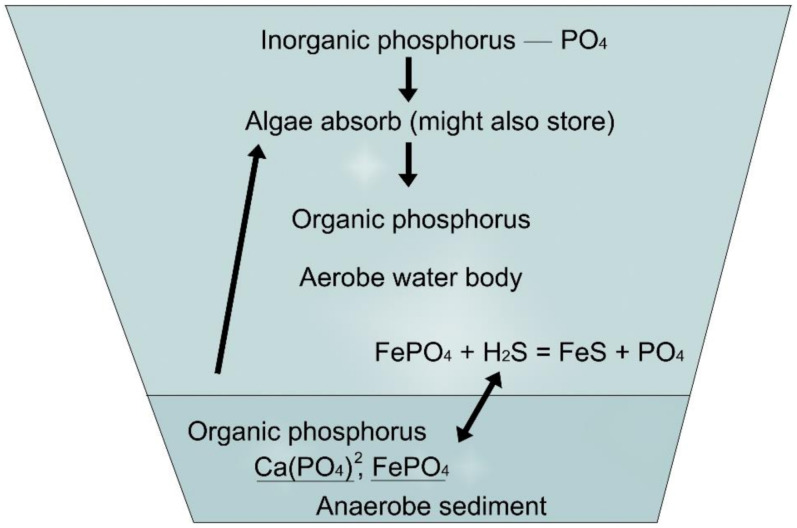
Phosphorus cycle.

The cycles of nitrogen and phosphorus compounds in ponds can be studied in Figure 15 and Figure 16.

### 2.6. Aquatic Habitats and Communities

Aquatic organisms inhabit the entire area of a water body, from the bottom to the surface, unlike organisms of terrestrial areas. The same organisms can live on different surfaces, or in open water as well (planktonic lifestyle); thus, aquatic communities and habitats occupied by them can be very diverse.

The most common freshwater habitats are:

- Open water area, called pelagic zone, which provides an environment for (a) floating communities of plankton and (b) a rapidly swimming biocenosis called nekton, composed mainly of fish species.
- The surface habitat, called facial, which has two communities, the moving pleuston with larger members and neuston with tiny ones.
- The vegetation-rich lake shore zone with a shallow water depth; the phytal and littoral zones have mixed communities. Periphyton lives on the surfaces of water vegetation, but in the littoral zone, members of the plankton are generally also present.
- Both the pelagic and littoral zones have sedimental bottom areas, which create a benthic habitat where members of a community called benthos live.

These habitats and communities (biocenosis) are present in artificially constructed fishponds as well.

### 2.7. Competition of Water Plants in Ponds

The next important and exciting question is: how the number of one-cell algae can be increased in shallow ponds, where they need to compete for carbon sources and nutrient elements with higher water weeds and cyanobacteria.

**Figure 17:**
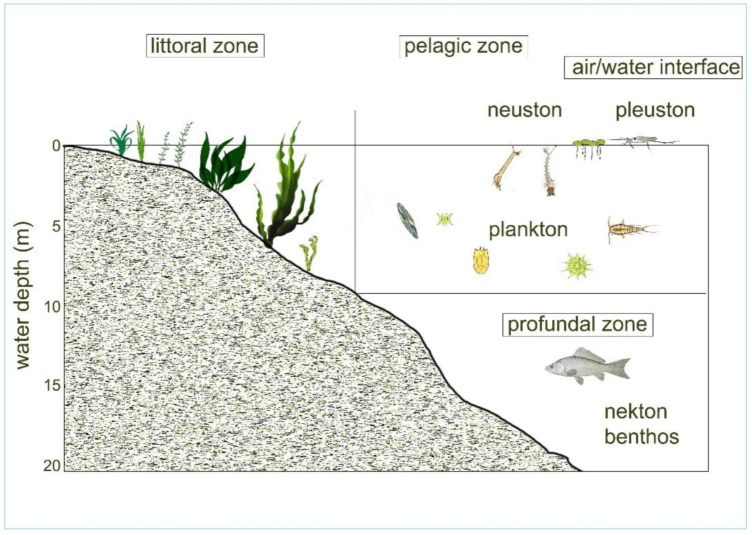
Most important habitats and communities in lakes/stagnant waters.

Water weeds in shallow waters can be divided into two groups: emersed and submerged types of plants (Figure 17).

Leaves, stems, shoots, flowers and seeds of emersed plants extend above water level, while their stems remain underwater and roots in the mud. During photosynthesis, carbon dioxide is assimilated from the atmosphere, while nutrients are obtained from the water and the sediment. Some of the submersed aquatic plants have roots as well, while others float freely in the water body. Moreover, there are higher aquatic plants that live at the water–air phase. Controlling these diverse groups in ponds is possible by either mechanical or biological methods. Emersed plants can mainly be controlled mechanically, while submerged water weeds, in addition to mechanical methods, can also be controlled efficiently via biological practices (mainly by stocking large grass carp). Increasing water turbidity can also be effective, as there would not be enough solar energy in turbid waters for assimilation.

Regulating the blooming of cyanobacteria (formerly mentioned as blue-green algae) is also an important and difficult task. Some species of these tiny aquatic plants consist of a single cell, while others form colonies. Certain species are also able to bind nitrogen from the atmosphere and therefore are able to reproduce in an environment with limited nitrogen availability.

In contrast to these groups, unicellular green algae, which are important for pond production, can only multiply rapidly and synthesize organic matter in water bodies with high nutrient content, and without the competition of other plants.

If it is possible to produce turbid lake water, solar energy will be absorbed in the upper 20–30 cm, heating up this layer and creating a suitable environment for the development of green algae. This upper layer is called the trophogenic zone, where green algae can multiply and fix solar energy into synthesized organic compounds. Organic substance and energy can migrate through the food chain towards higher trophic levels. In turbid waters, neither submersed macrophytes nor dangerous cyanobacteria living on the bottom of shallow ponds receive enough solar energy.

**Figure 18:**
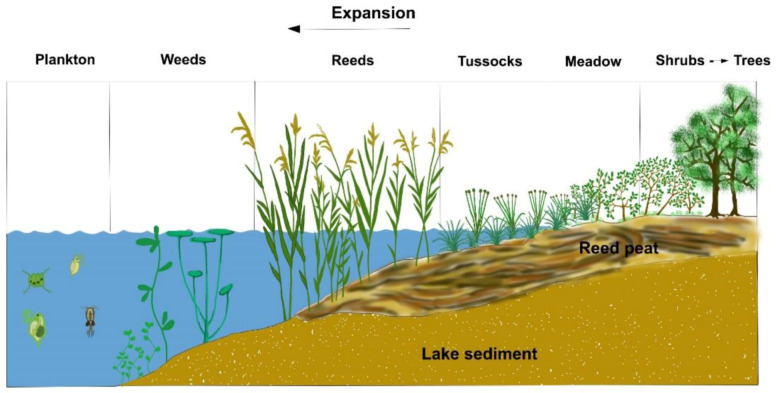
Zonation of macrophytes from open water bodies containing algae to forests.

**Figure 19:**
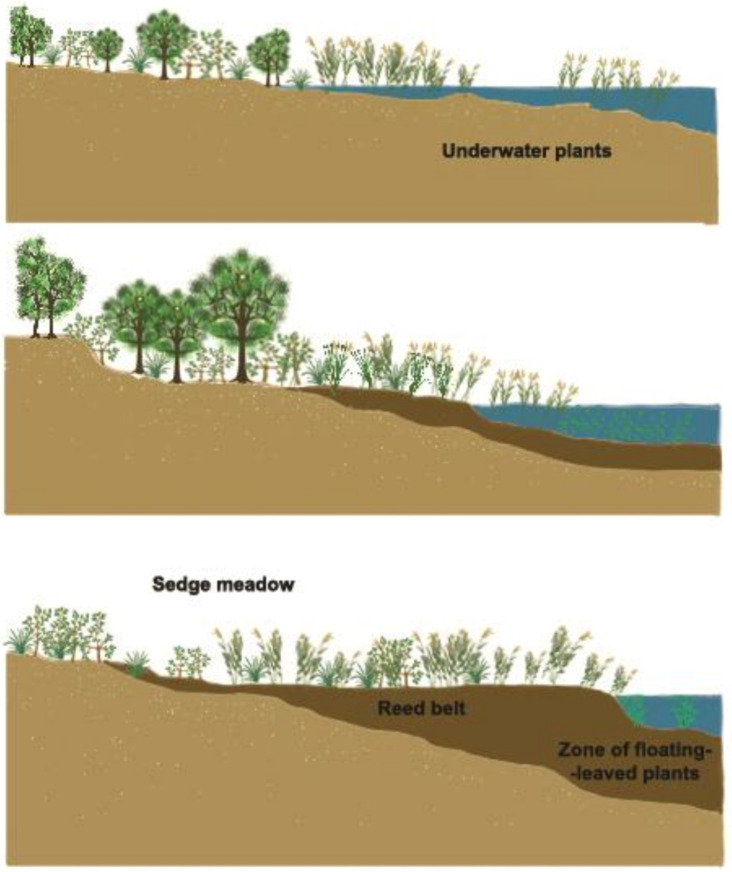
Proliferation of the macrovegetation enables the growth of organic sediment and the recharge of shallow fishponds.

**Figure 20:**
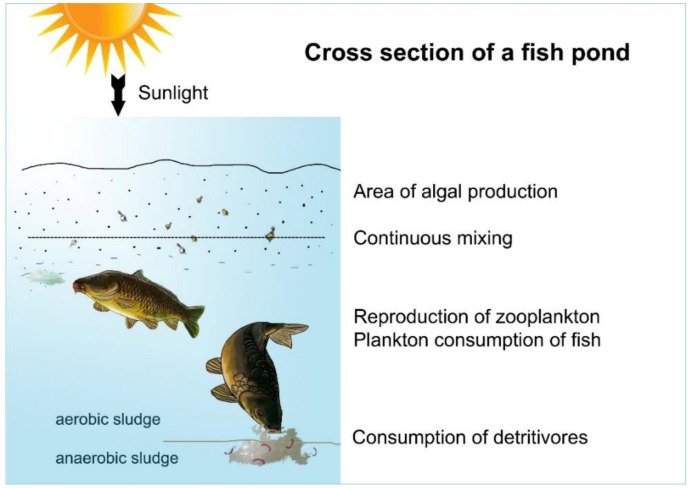
Cross section of a shallow pond. The upper part of the water, where intensive algal assimilation happens, is the trophogenic zone. Carp feed on zooplankton from open water or from mud sediment.

Carp bioturbation is the most effective way to disturb pond water (see Section 1.3). Furthermore, a regular mechanical agitation of settled organic sediments and a frequent organic fertilization and liming of the pond water also have a positive effect on disturbance. Figure 18, Figure 19 and Figure 20 demonstrate the cross-section and activities of a shallow fishpond.

### 2.8. The Origin of Carbon Dioxide Required for the Synthesis of Organic Matter in Fishponds

Organic matter synthesized by algae requires large amounts of carbon dioxide. To form one molecule of a simple sugar, six molecules of carbon dioxide are required. Organic macromolecules such as starch or cellulose are composed of a large number, sometimes even many thousands, of simple sugar molecules. For the synthesis of proteins and fatty acids, not only carbon dioxide but also nitrogen and phosphorous compounds are needed. In addition to these basic substances, many other organic molecules, such as hormones, vitamins, enzymes, DNA and RNA macromolecules, existing in living organisms, need carbon dioxide (hydrogen carbonate within aquatic conditions). There was a theory in the past that carbon dioxide was a limiting factor for biological production. This is absolutely true for the so-called oligotrophic, nutrient-poor, cold and deep natural lakes, but not for shallow, nutrient-rich eutrophic fishponds, where carbon dioxide can emerge from many sources. A distinction can be made between carbon dioxide from endogenous/internal and exogenous/external sources.

### 2.9. Carbon Dioxide Originating from Endogenous Sources

An important endogenous source for carbon dioxide is nocturnal dissimilation or “respiration” of pond biocoenosis, another one is the metabolism of algae, cianobacteria, higher water vegetation, aerobic and anaerobic water bacteria, invertebrates and fish. The amount of metabolic of carbon dioxide can be deduced from the difference between the maximum and minimum daily levels of dissolved oxygen generated as a by-product of assimilation on the previous day (for details, see Section 2.5) The basis of this calculation is the monitoring of oxygen production and consumption in proportion to the magnitude of assimilation and dissimilation.

Another internal source of carbon dioxide in the lake is the decomposition activities of settled organic particles (detritus) by water bacteria and tiny fungi, as well as by invertebrates (e.g., chironomids, worms and snails). After the extinction of aquatic macrovegetation (in the autumn), a lot of organic matter enters the sediment, which immediately begins to decompose. Aerobic decomposition occurs in the upper part of the sedentary organic matter, because oxygen from the oxygen-rich water body diffuses into the sediment. The result of this decomposition is carbon dioxide, which is instantly converted to a hydrogen carbonate ion which bonds ionically to a cation present, or forms carbon dioxide gas bubbles in the sediment. In deeper layers of the sediment, within reducing conditions, not only does carbon dioxide develop as a product of decomposition, but methane gas as well, which either effervesces through water or forms bubbles in the sediment. When methane is released from the sediment (as air pressure decreases), it removes dissolved oxygen from the water body while methane is oxidized to carbon dioxide.

Further important endogenous carbon dioxide sources originate in recycling, resuspending dead, sedimented organic matter accumulating in the mud into oxygen-rich water space. Aerobic decomposition of the recycled organic sediment produces a large amount of carbon dioxide. This process is initiated by the resuspending activity of pond organisms, especially of common carp and grass carp. The food search of these fish in the sediment induces a strong bioturbation. Not only fish but also many invertebrates, such as worms, snails, mussels and larvae of insect living in the mud, resuspend the sedimentary organic detritus. In addition to the bioturbation of living organisms, agrotechnical methods can also be applied (e.g., sludge blasting, sediment mixing). Processes that facilitate the carbon release from the sediment partially eliminate or diminish this “dead end” of organic matter.

### 2.10. Carbon Dioxide Originating from Exogenous Sources

The origin of exogenous carbon dioxide is even more diverse.

When inundating ponds in spring or adding extra water in summer, the water entering to the pond can transport a lot of inorganic carbon (carbon dioxide, bicarbonate, carbon anions) and dissolved or particulate organic matter where algae immediately begin to absorb the inorganic carbon via osmotrophy, and bacteria start to decompose detritus to carbon dioxide.

The next source of CO_2_ is the atmospheric gaseous carbon dioxide entering lake water via diffusion, as substances with different concentrations tend to level off. The concentration of carbon dioxide in the air is 0.03%, which is constantly increasing due to global warming. As discussed earlier, in well-buffered neutral (Ca/Mg rich) waters, carbon dioxide is present in the form of hydrocarbonate. There is practically no free carbon dioxide in ponds, so due to the equalization of partial atmospheric pressure, CO_2_ slowly diffuses from the air into the water. Calcium carbonate crystals (calcite) in water immediately react with carbon dioxide immigrating into the water and are converted to hydrocarbonate anions. Seawater (with limited Ca/Mg cations and dominating Na/K cations) only has a weak Ca/Mg hydrocarbon/carbonate buffering system. In other words, calcium carbonate crystals cannot absorb carbon dioxide; thus, they form hydrocarbon anions (see Figure 6). Carbon dioxide is bound to hydrogen ions and forms a weak acid called carbonic acid (H_2_CO_3_), which is responsible for the slow acidification of oceans. The same process can be observed in freshwater bodies on volcanic soil, which contain little to no Ca/Mg cations.

**Figure 21:**
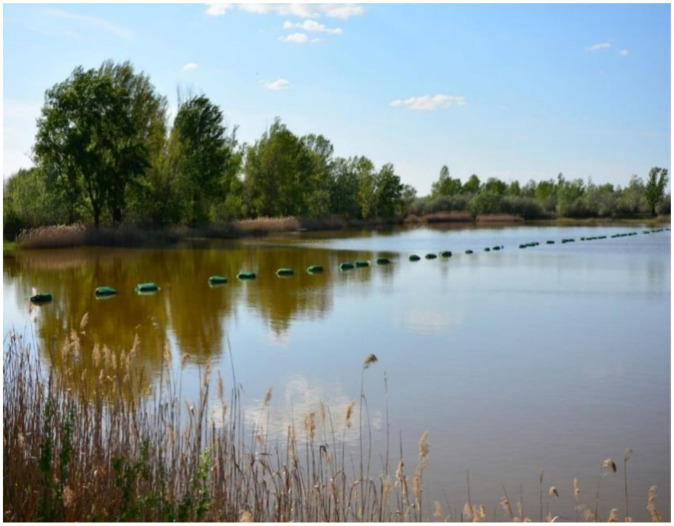
Raschel bags filled with straw reduce the growth of cyanobacteria (Photo: A. Hegyi).

Another exogenous source of carbon dioxide can be an efficient agricultural intervention, when dry straw with a huge amount of cellulose is transferred to the ponds. This treatment (Figure 21) controls the reproduction of cyanobacteria and stops their blooming. The blocking effect of straw is the result of its polyphenol compounds diffusing into the water, which is only toxic to blue green algae (cyanobacteria). Within a few days, this effect eliminates cyanobacteria and a new controlling mechanism develops instead: a strong nutrient competition between aquatic microfungi and cyanobacteria. An aggressive microfungi population starts to develop on the high carbon content of straw which takes up all N and P components of water; therefore, cyanobacteria starve. Under these circumstances, a new food chain occurs: rotifers and ciliates feeding on microfungi. These planktonic organisms are consumed by copepods, which are then filtered by carp, thus forming a five-step food chain in ponds. The cellulose from straw practically appears as an exogenous carbon source in ponds.

All agrotechnical treatments of the breeding protocol (fertilization with organic manure, fish feeding and pond liming) can be evaluated as large amounts of important exogenous carbon dioxide sources. Carbon dioxide is produced from organic manure through the same decomposition processes as from the sediment discussed in the previous sections.

**Figure 22:**
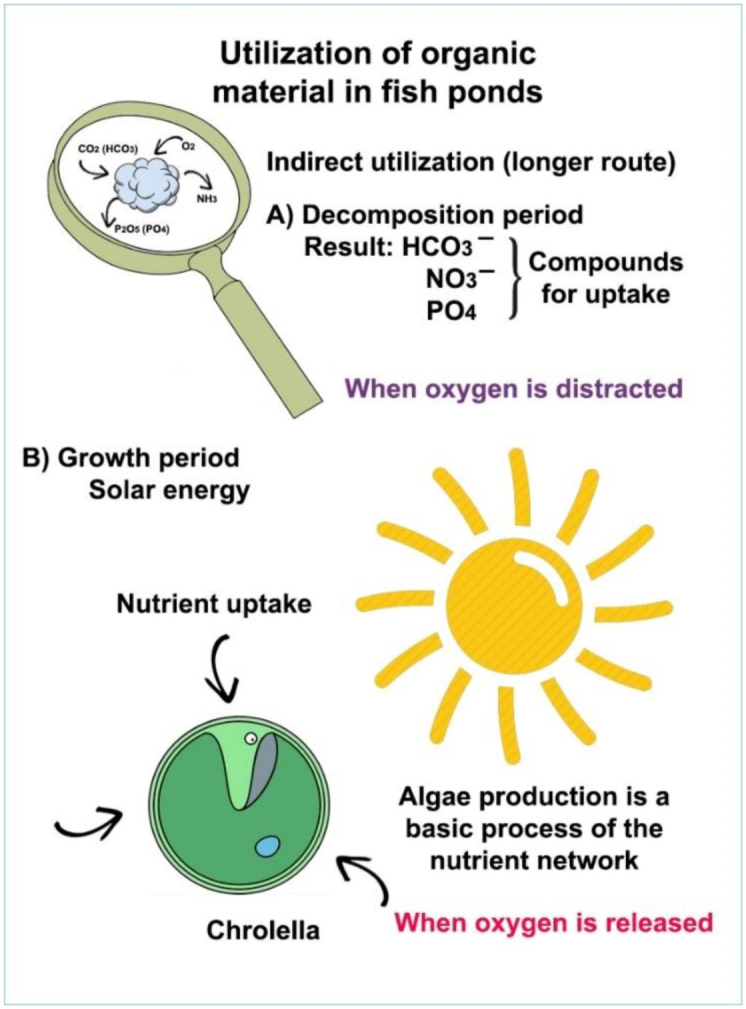
Two adverse processes of organic compounds in the ponds: (**A**) decomposition of dead organic matter to carbon dioxide; (**B**) production of new, energy-rich organic compounds by algal assimilation.

During the decomposition process of organic manure (Figure 22), not only is carbon dioxide produced but also many other elements and compounds (molecules with N and P content) which play an essential role in the reproduction of algae; therefore, mature animal manure has a complex effect on biological production.

A continuous enabling of the uptake of carbon dioxide and other important biogenic elements for algae has created suitable conditions for continuous reproduction. However, some questions still remain open: what happens with the algal biomass? How will high-quality freshwater fish products suitable for human consumption be developed from the primary producer water algae? To answer this question, the whole process of energy transfer and trophic levels in a pond ecosystem needs to be analysed. The synthesis of inorganic carbon compounds into organic matter is the result of plant assimilation. Newly produced organic matter (biomass of algae) enters the food chain when consumed by heterotrophic organisms.

### 2.11. Trophic Levels in Terrestrial and Aquatic Ecosystems

According to a simple definition, an ecosystem is a self-regulating biotic community (biocenosis) together with its physical and chemical environment, where interactions occur between the organisms and the environment. The migration and transformation of all organic matter/energy in an ecosystem is triggered by vital activities of biological organisms. The most important activity within the interactions of living organisms of an ecosystem is consumption (trophic levels). While osmotrophy is the feeding mechanism of autotrophic plants to take up nutrient elements, animals use phagotrophy for that. The organisms use the energy of nutrients/foods for metabolisms and to increase their biomass.

**Figure 23:**
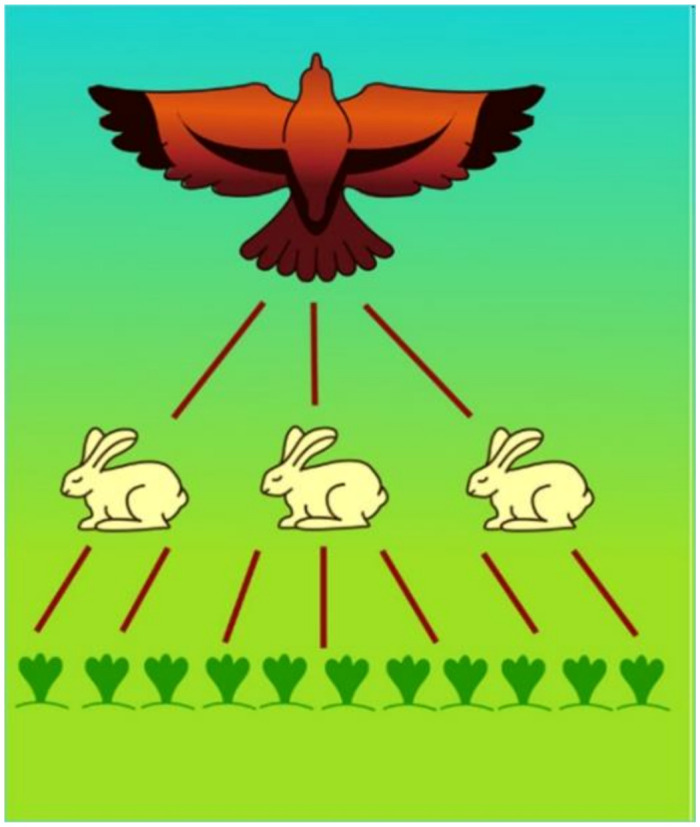
Progress of organic matter and its energy through trophic levels: autotrophic plants as Primary producers, plant consumers as Primary consumers, predators as Secondary consumers (in this figure: top predator).

Energy-rich organic matter can only be produced by autotrophic plants. The migration pathways of organic matter and stored energy via the different trophic levels were recognized by the founder of modern ecology, Charles Elton (1900–1991), an ecologist from Oxford. His discovery [25] is modelled in Figure 23.

This ecological law is not only valid for terrestrial ecosystems but for aquatic one as well. 

**Figure 24:**
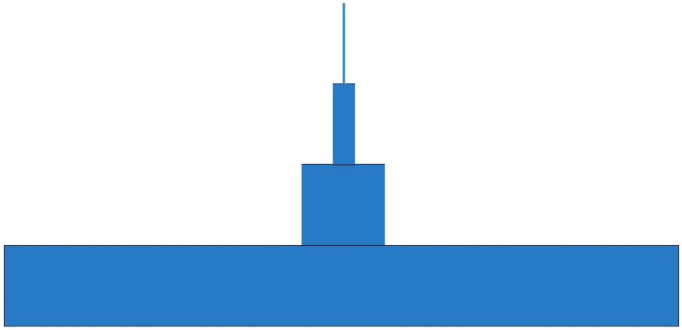
Quantitative relationship of different trophic levels in a lake ecosystem. From bottom to top: algae, fish consuming zooplankton and algae, fish consuming zooplankton.

The efficiency of biomass incorporation between different trophic levels is rather poor at no more than 10%[26]; therefore, the organic energy of primary production (algae) is dissipated via a maximum of four trophic levels (see Figure 24).

**Figure 25:**
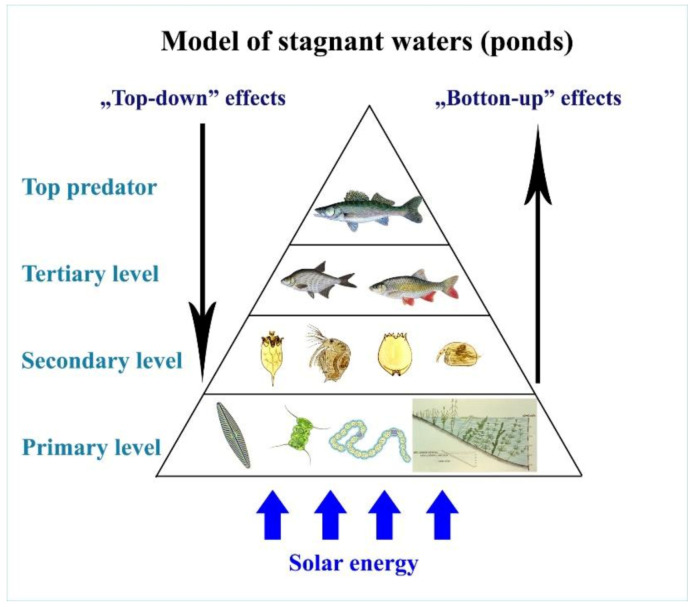
A food chain with four levels in a natural lake.

**Figure 26:**
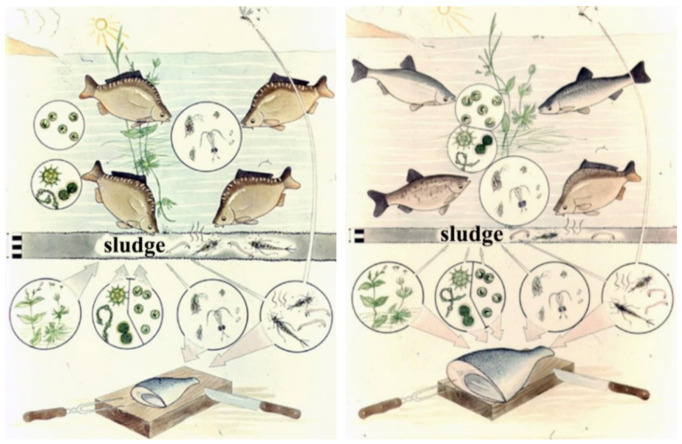
Fish production ratios based on natural food: monoculture vs. polyculture.

Quantitative ratios of fish products based on natural (evolutionary preference) food are shown in Figure 25. In the classical Central-Eastern European production system, carp is a dominant species. It belongs to the tertiary trophic level and consumes zooplankton and zoobenthos from the secondary trophic level. In Asian multispecies cultures, which are economically more productive, carp is stocked together with herbivorous fishes. The Asian fish species of the second trophic level consume algae from the primary production level and higher water vegetation as well. Therefore, based on Figure 26, the total fish biomass produced in polycultures can theoretically be ten times higher than that of a carp monoculture. Unfortunately, the population of Central and Eastern Europe, which traditionally consume carp, is not open to accepting fish products made from herbivorous fish, which are produced much more cheaply.

Investigating the route of the food chain and energy transfer in pond farming systems dominated by carp, at first sight it seems to be evident that the process of energy transfer is very similar to that of natural lake ecosystems (see Figure 24). The main difference is the huge number of stocked fish in fishponds. Consequently, the migrating energy and the total biological production are much higher in them. When stocking predator fish in ponds, the goal is not the consumption of carp but the elimination of invasive trash fish that are food competitors for carp.

**Figure 27:**
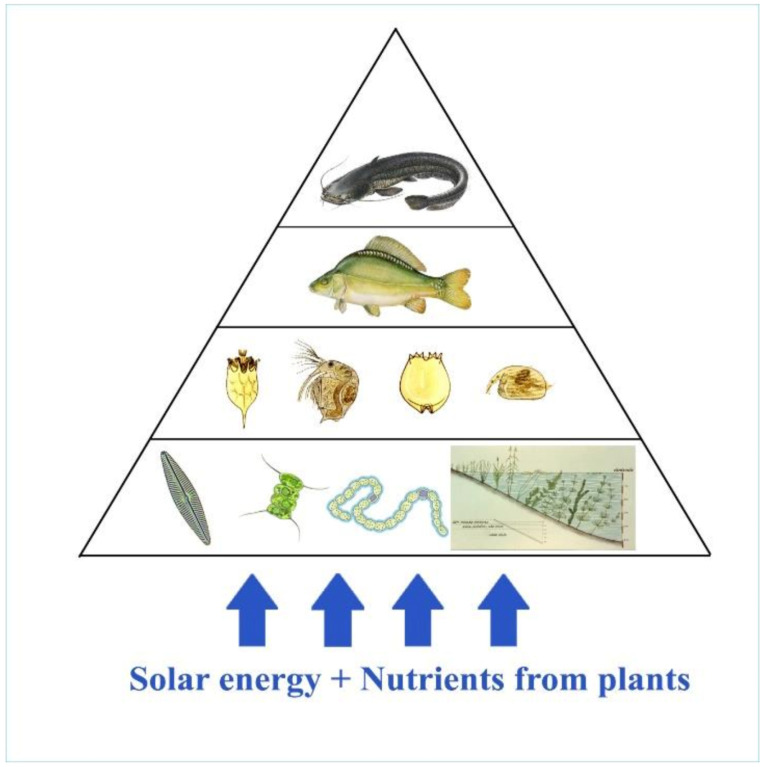
At first sight, trophic levels in ponds are very similar to that of natural lakes. However, huge differences can be detected due to the number of stocked fish.

A deeper investigation of the circulation of carbon compounds in a fishpond shows significant differences, thanks to the special feeding habit of carps stocked in large numbers. First the bottom-up regulation of energy transfer in ponds is analysed, as presented in Figure 27.

**Figure 28:**
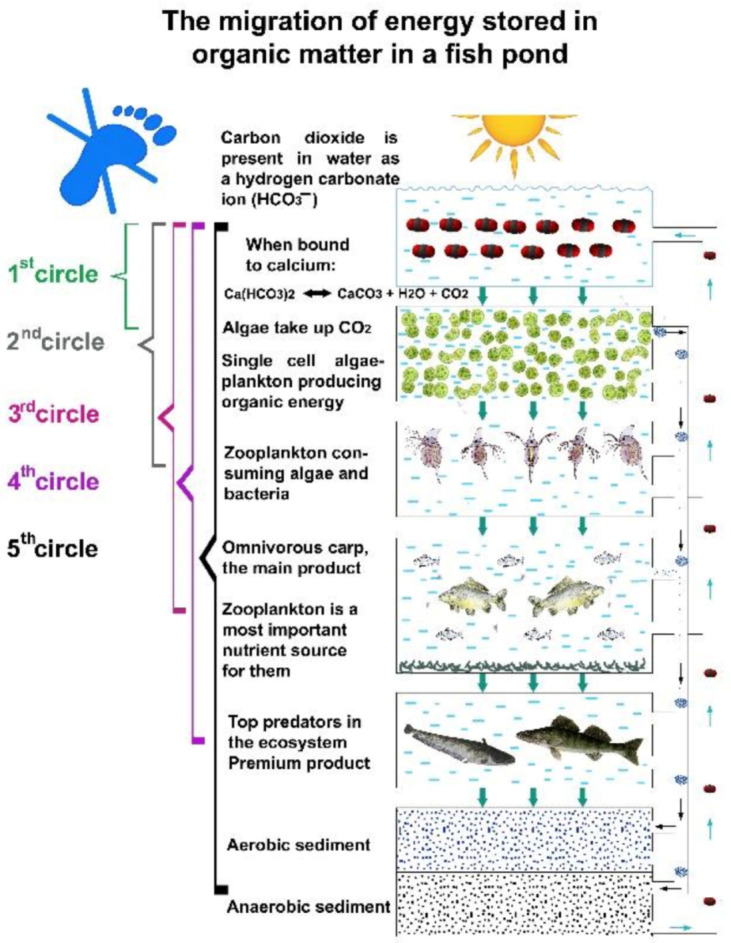
The migration of carbon atoms via trophic levels of pond ecosystems. Energized carbon compounds produced by algae start to move to upper trophic levels. Between two levels, the loss of energy is around 90%.

**Figure 29:**
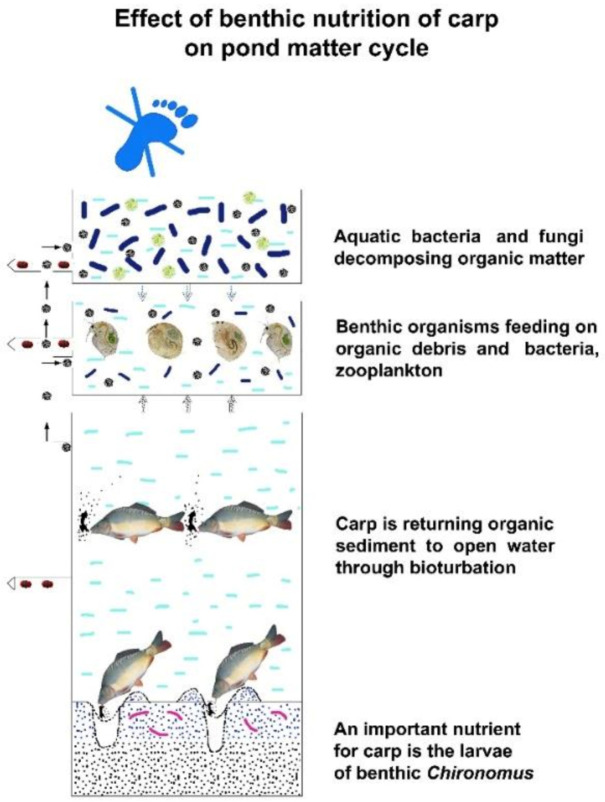
Bioturbation of common carp in pond culture systems. Organic mud is resuspended forming a trophogenic layer and a production environment.

At each trophic level, a small part (10%) of the energy fixed in carbon compounds by algae integrates into organisms—biomass—of the next level, while a much greater part is used to cover the metabolic energy needs of organisms of the current level. This larger part of the energy dissipates from the system. The final product of metabolic activities (dissimilation) is carbon dioxide (present in water as bicarbonate), which then returns to the first nutrient level (carbon circles 1–5), and after being reenergized by algae, the carbon migration process starts again. As Figure 28 indicates, this type of biological production has practically no carbon footprint, as carbon dioxide cannot leave the system (staying in the form of bicarbonates) but circulates in the water until being trapped either as fish biomass or as organic sediment in the mud. This migration of biological matter and energy in fishponds is very similar to the process of energy transfer in natural eutrophic lakes. However, when carp feeding habits are analysed, significant differences can be observed. The large number of carp present in ponds (and not in lakes) can modify the physical circumstances. As discussed in Section 1.3., common carp not only filters zooplankton, but also feeds on worms, snails, etc., from sedentary mud. When a large number of carp hunt these organisms with a high protein content, it results in a resuspension of organic mud (called bioturbation). The consequence is a provoked, quick carbon circulation, eliminating or at least decreasing the amount of carbon trapped in the sediment and increasing carbon fixation in fish biomass. This is a notable advantage of carp culture systems: the large number of carp influences the production environment by developing a significant trophogenic zone for algae production and also a quick carbon circulation within a pond without any carbon footprint (see Figure 29).

To summarize the above analysed chemical and biological processes of water, it is evident that, in fishponds, electron pairs in covalent and ionic bonds keep carbon dioxide molecules in stable forms within bicarbonate (HCO_3_) and carbonate (CO_3_) anions; therefore, gaseous carbon dioxide is not able to diffuse into the atmosphere, nor can it increase the amount of atmospheric greenhouse gases, which is favourable from an environmental point of view.

## 3. Pond Breeding Technology in Central Europe

From a hydrobiological point of view, fishponds in Central Europe, belong to the group of artificially made, shallow, freshwater ponds which exist at a temperate climate. Water quality is considered to be optimal when it is characterized by a high amount of Ca/Mg cations and CO_3_/HCO_3_ anions.

In these specifically constructed production units, the properly controlled technology provides a profitable product, a well-marketable freshwater fish. The breeding technology contains fully detailed technological steps or so-called agrotechnical interventions. Fish stocks utilize essential proteins and fatty acids of renewable biological production, zooplankton and zoobenthos, supplied with energy-rich grains provided by fish farmers as supplementary feeds. 

**Figure 30:**
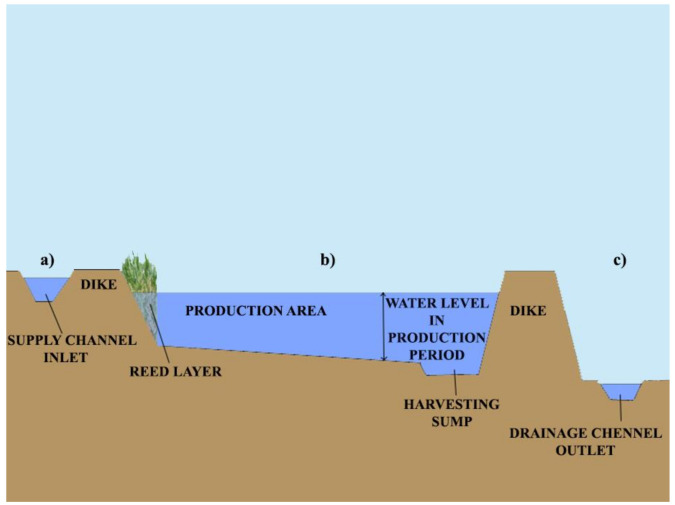
Favourable slope conditions of a fishpond: (**a**) supply channel/inlet; (**b**) water level in the production season; (**c**) drainage channel/outlet.

**Figure 31:**
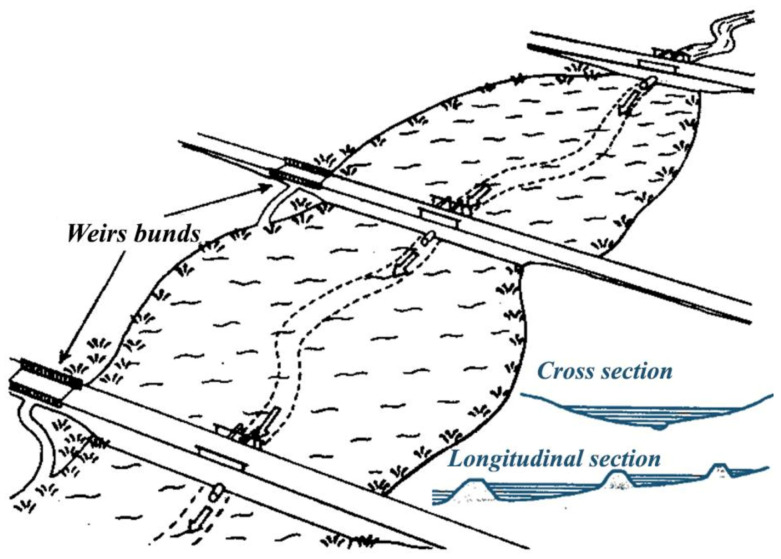
Series of lakes between low hills: the water in the valley is separated into lakes by cross ditches.

**Figure 32:**
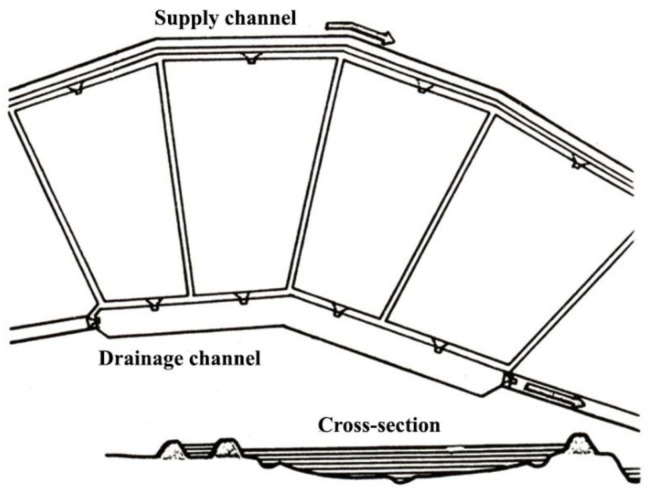
A group of ponds in a flat area. Shared fishing grounds and stable road conditions help autumn fishing. Below is a cross-section.

For profitable fish production, properly planned and constructed fishponds (Figure 30, Figure 31 and Figure 32) are needed, where agrotechnical treatments can support the healthy growth of fish. Constructed breeding ponds have some special technical units, such as monks, supply and draining channels, overflow units, inside or outside harvesting places, well-built roads to fishing places, fish-selecting places, temporary places to store fish with a constant water flow to secure additional oxygenation, etc.

A very important technical requirement is the source and amount of water to fill up ponds in spring and restore water in summer. It is beneficial to have a water source that is regulated by gravitation.

### 3.1. Advanced Breeding Technique Applied under Temperate Climate in Shallow Fishponds

Whole-year fish breeding activities can be divided into two main categories: (A) active growing season in the summer and (B) an inactive period in the wintertime.

**Figure 33:**
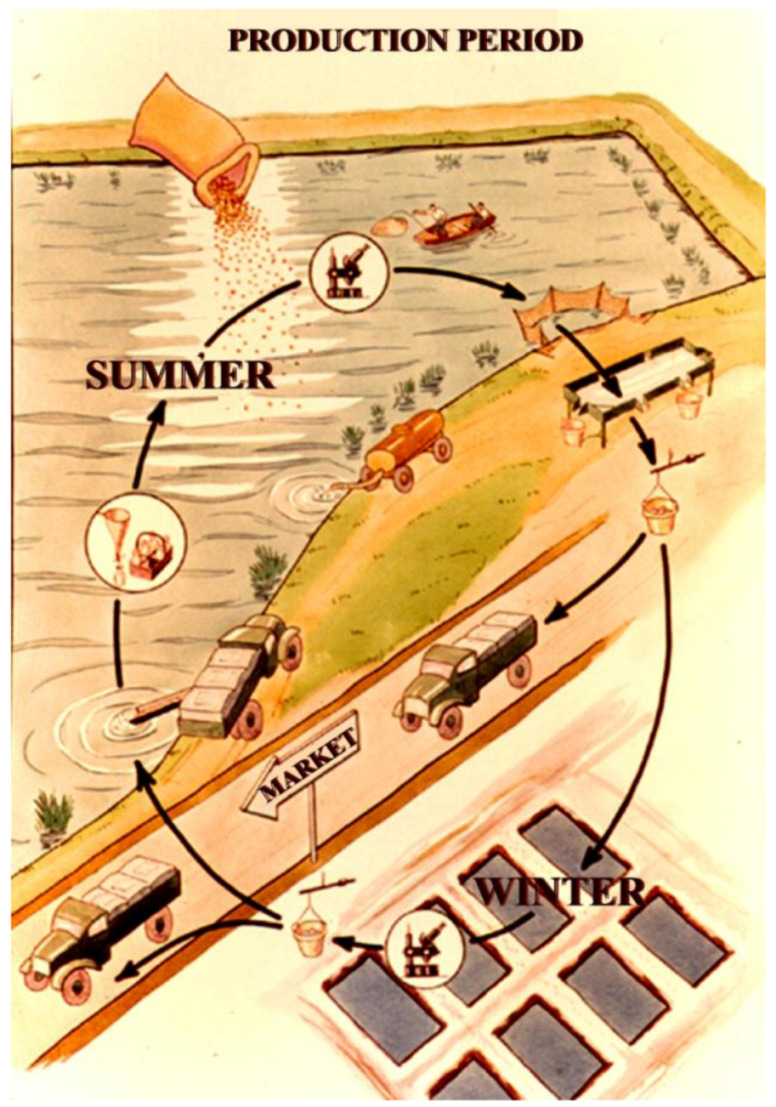
Seasonal periods of a year-round breeding system.

A year-round breeding technology can be examined in Figure 33, where all important agricultural treatments are indicated following each other [8,16,17,20].

The summer season (A) starts with pond preparation: removing vegetation from the previous season, filling up the pond with filtered water, introducing nutrients (organic manure) to feed zooplankton and the application of preventive liming, if necessary. These activities are followed by stocking fish into the ponds after careful transportation, starting supplementary feeding with cereals, health control by sampling the stock systematically, managing fish development and, finally, removing them from growing ponds at the end of the production season (autumn).

The wintering period (B) starts when selected fish stocks are transported from autumn fishing grounds to special wintering ponds. This period is somewhat simpler than the busy summer period. In this term, the main tasks of fish farmers are to keep the fish stocks in a good condition, take care of them and control them. During wintertime, fish stocks live in small ponds. The number of fish in wintering ponds is very high; therefore, the stocks need a continuous water supply to eliminate waste metabolic compounds. Besides that, continuous water inflow also transports dissolved oxygen to fish, which have a low metabolic activity (with basically no feeding) in cold water. Wintering fish stocks are periodically investigated by a veterinary expert to prevent infections and, if necessary, treat the stocks to avoid losses caused by diseases.

In the following, separated technological sections are overviewed and discussed.

**Figure 34:**
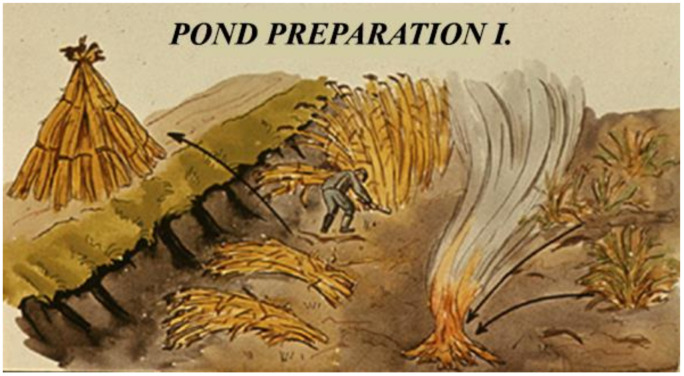
Cleaning the pond from remains of dried water vegetation.

**Figure 35:**
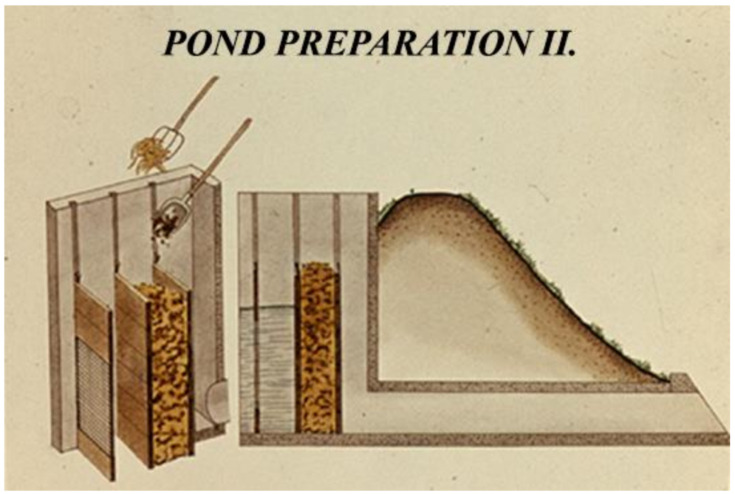
Cross-section of a dam and a filling (water-level regulating) monk. First, the necessary water level is adjusted by removable boards. At the first line, filtering nets are applied to prevent the entry of invasive fish populations. During the filling of ponds, filter grilles need to be cleaned from time to time.

The growing season starts with the preparation of ponds, including the elimination of the dry remains of water vegetation (Figure 34) and the reparation of damaged dams (Figure 35).

Pond cleaning is necessary as harmful parasites can overwinter on the dry parts of reeds, etc. On the other hand, vegetation growing on embankments and dams plays an important role in the summer, as their roots and biomass provides protection against the degrading effect of strong waves generated by winds.

**Figure 36:**
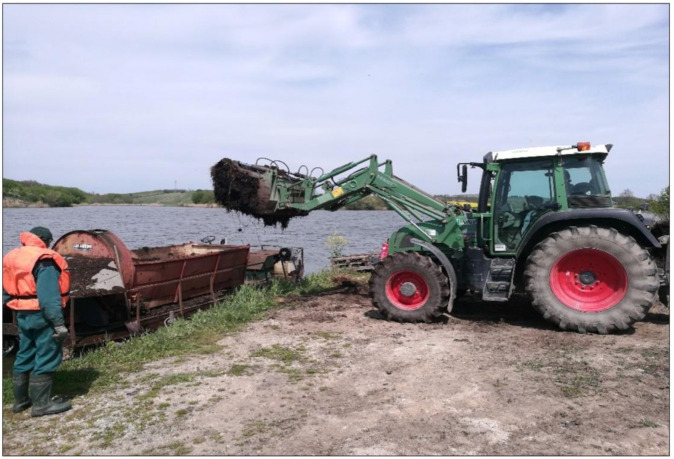
A weight-lifting tractor transports wet or semi-dry organic matter into a large boat (Photo: A. Hegyi).

**Figure 37:**
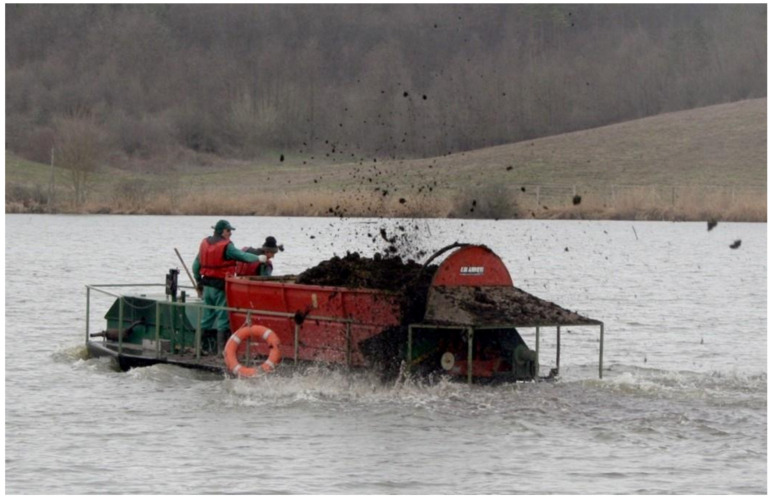
Specifically constructed shredding equipment, the so-called “manure-spraying machine”, helps to cut the large particles of manure into smaller ones, thus increasing the surface for a fast bacterial decomposition. Some of the small organic particles (detritus) are directly utilized as food by members of the zooplankton and zoobenthos.

**Figure 38:**
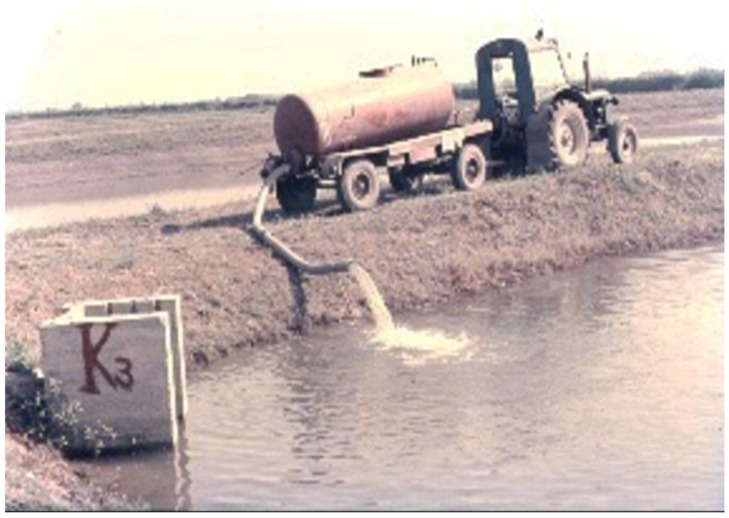
Liquid manuring of a nursing pond. Dissolved organic compounds decompose rapidly; therefore, the biological effect is quick.

At large fishponds, the filling process lasts for several days. When the water in ponds reaches an optimal depth of growing (“summer level”), the pond preparation continues with the introduction of nutrient elements. Normally, organic manures are used for this purpose; after bacterial decomposition, it supports the reproduction and biological production of one-cell algae, which serves as food for zooplankton. The amount of required organic manure for one hectare is 2–7 metric tonnes/season, which should be applied in smaller portions. During spring, when the pond is under preparation for the growing season, half of the yearly amount is released. The other half should evenly be distributed during the production season. To facilitate treatment with heavy, wet organic manure, the process is mechanized (Figure 36, Figure 37 and Figure 38).

The most efficient way of organic manuring is when the manure is transported by a large boat (Figure 36 and Figure 37) and is released from there into the water of the pond.

It is important to specify again that the application of organic manure does not serve as direct food for fish, but it increases the biological production of pond ecosystems. According to field experiences, every hundred kg of semi-dry organic manure will result in 3–4 kg of extra carp production.

Well-managed pond preparation results in an optimal environment for fish populations to ensure a profitable growing season, so that the fishpond is ready to accept fish.

The first step of releasing fish into production ponds is to remove them from wintering ponds.

**Figure 39:**
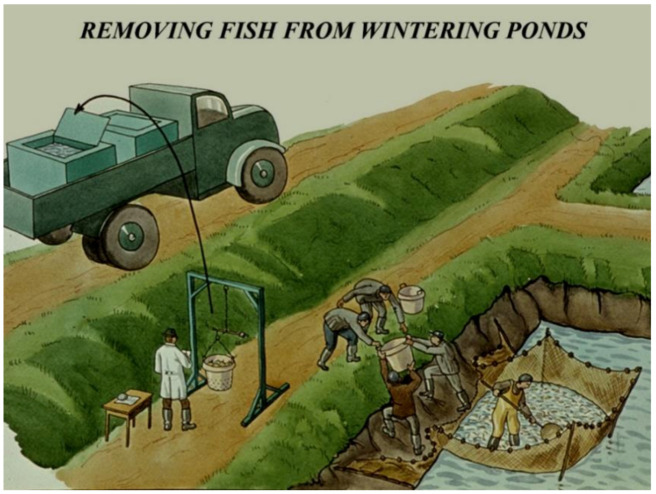
Fishing from wintering ponds; placing fish into trucks or camions with special fish transport tanks and oxygen supply.

To start this procedure, fish are collected by netting them carefully (Figure 39), then their weights are measured, and finally, they are put into transporting cars with plastic basins. During transportation, fish are in a high density; therefore, they are continuously in need of extra pure oxygen.

**Figure 40:**
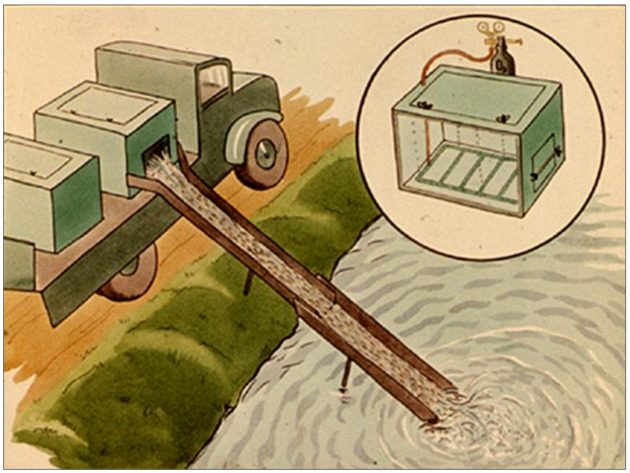
Transported fish stocks are carefully released into a pond using a plastic trough.

When the transport car with fish arrives to the growing pond (Figure 40), the first parameter that needs to be checked is the temperature. A large difference between the temperature of the transporting water and pond water can cause a heat shock; therefore, temperature equalization is necessary.

In general, the period of pond stocking is during spring (March–April, depending on the weather), when the water temperature is around 8–12 °C. This water temperature is still too cold for an effective metabolism, as carp need warmer water for active feeding (over 15 °C). Cold water during early spring does not provide optimal feeding conditions; thus, the intensive feeding of carp is economically not viable, as some of the food remains on the feeding ground and the expensive cereals gradually degrade. Therefore, following the spring stocking, a so-called supplementary feeding is applied with careful attention and continuous control.

### 3.2. General Roles of Supplementary Feeding

In a traditional breeding structure, carp in temperate climates need three seasons to reach market size (1.5–2 kg/fish). The first year lasts from the hatching of the new generation in May–June up to the end of the production season (September–October). This is followed by a wintering period that lasts till next March–April. During the first growing season, the average stocking density is about 50,000–100,000 fish per hectare, and the losses are around 30–40%, depending on the number of predators. The end weight of fingerlings is 20–40 g/fish. In this intensive growing season, carp fingerlings need 40–45% of complete, digestible protein. In the second season, the stocking density is about 5000–10,000 fish per hectare, losses are approximately 20–30% and end weight is roughly 200–300 g/fish. The protein need of the fish is 35–38%.

In the third season, the carp density is 1200–2000 fish per hectare, losses in the season are 10–20%, the digestible protein need is 28–30% and the market size of fish is 1.5–2 kg (Table 1).

**Figure 41:**
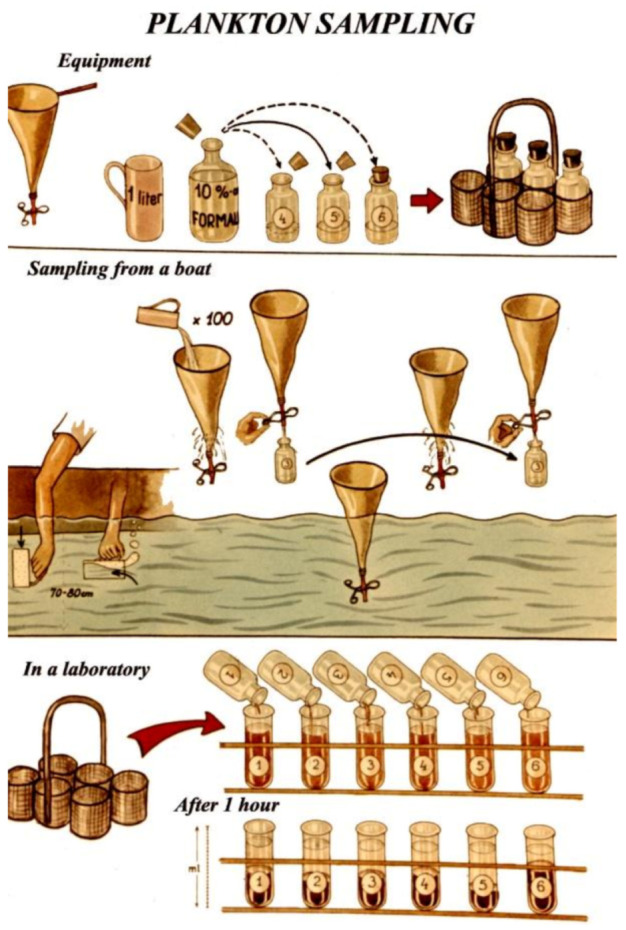
The process of zooplankton sampling.

If fish producers have their ponds well prepared for the growing season, by the time of spring stocking, every hundred litres of pond water contain at least 1–3 mL of live zooplankton biomass. To determine the biomass of zooplankton pond, sampling is required (Figure 41), for which a well-constructed plankton net is necessary. Its suggested mesh size is about 100–120 μm. Through this plankton net, all zooplankton organisms are filtered from the water. Moving diagonally through the pond, a hundred litres of pond water should be taken with a 1 L bottle from different places and transferred trough the plankton net. At the end of the sampling process, the filtered biomass is released into a small glass tube, then a few drops of 4% formaldehyde are introduced into it, which due to its toxic effect, kills all planktonic organisms. Within minutes, the mass of it starts to sediment in the tube, and the wet volume can visually be determined. This field sampling method only provides semi-scientific data of the zooplankton biomass, but that is good enough to estimate the protein source available for carp feeding.

Following spring stockings, an important question is when and how much feed to give to fish stocks per day.

**Figure 42:**
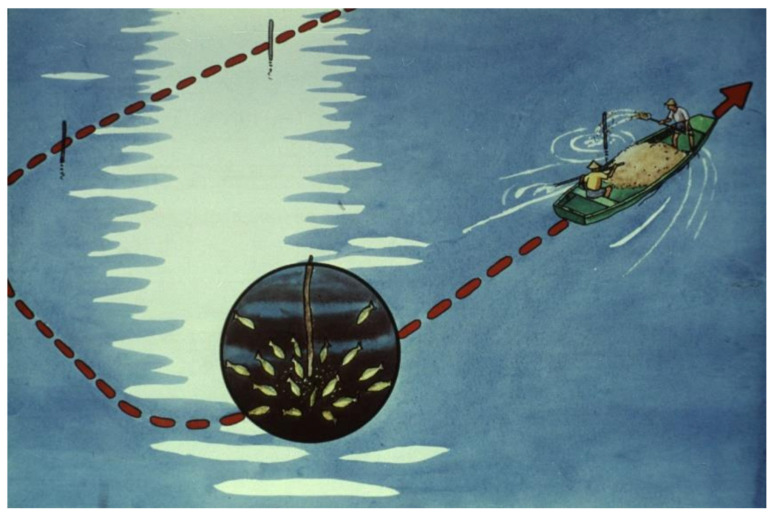
Feeding points in a pond. The grain is transported by boat.

At the beginning of the growing season, intensive feeding is not suggested. The water temperature is too cold for carp, and they need some days to adapt to the new environment. As a first action, before feeding is started, standard feeding points should be marked with a stick within the pond; 3–5 sticks can be fixed in every hectare (Figure 42).

**Figure 43:**
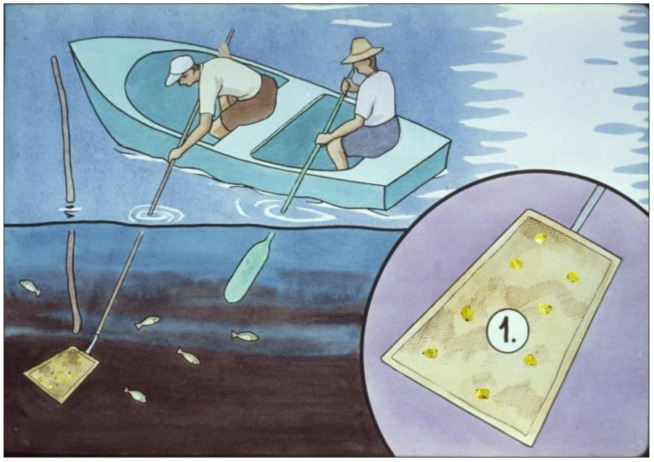
Grain consumption of fish must be monitored following feeding. (1) Some seeds can still be found on the research frame.

Feed, which is mostly different grains, is transported by boat to the feeding points. Feed consumption should be regularly monitored (Figure 43). The first inspection happens 3–4 h after the feed is released. If no feed is found, the amount of feed for the next day can carefully be increased. If there is still feed on the ground after 3–4 h, the inspection can be repeated after 7–8 h as well. If there is some feed left even then, then the next day the dose should be reduced.

Fish farmers can basically choose from two feeding strategies:

(a) Ad libitum feeding:

This strategy is based on a method that, regardless of the amount of natural food, fish farmers provide as much supplemental feed to their fish per day as they are able to consume.

(b) Feeding strategy based on the protein needs of different age groups of carp:

In an advanced method, protein of zooplankton origin is also considered when calculations are prepared. The zooplankton sampling process of pond water is presented above. To provide a better understanding of the method, a simple example is offered on a production pond of three-year-old carp, where the average weight of fish is 1000 g and the density is about 500 fish/ha. In this case, the total fish stock/ha is 500 × 1000 g = 500,000 g = 500 kg/ha. A single fish can consume a total amount of feed of about 3% of its body weight per day, so feed in 1 ha should be 15.0 kg/day. From this amount of food, 28% is needed by carp as digestible protein: 28% protein × 15 kg total feed means that 4.2 kg protein/ha is required. The 500 kg fish can have a maximum of 3% grains/kg body weight of fish, so 15 kg of grains with 11% digestible protein equals 1.6 kg of incomplete protein of grain origin.

On the other hand, calculations can also be made on behalf of zooplankton protein. A total of 500 fish with 1000 g individual fish weight means 500 kg fish/10,000 m^3^ (if general water depth is 1 m). Theoretically, each fish can filter 20 m^3^ of water. In the zooplankton sample, 100 L of pond water includes, e.g., 2 mL wet sedentary zooplankton, which means that 20 mL/m^3^ of zooplankton is available for fish consumption, which is 10,000 × 20 mL = 200,000 mL or g of wet zooplankton/ha in the investigated pond. Based on practical experiences, half of the amount of zooplankton is filtered by one fish each day, which in this case is 100,000 mL or g, or 100 kg/500 fish, so in theory, one fish is able to filter 200 g zooplankton/day. According to laboratory data, the dry matter of zooplankton is about 10%, from which the protein content is 60%. Therefore, 200 g × 0.10 × 0.60 = 12 g of complete protein is collected by each carp every day.

At population level, 500 fish × 12 g is 6000 g of high value proteins with zooplankton origin/ha, which are produced by renewable sources each day. To summarize, 2 mL zooplankton/100 L of pond water and 3% supplementary grain are able to provide 6 kg protein of zooplankton origin plus 1.6 kg protein of grain origin, which adds up to 7.6 kg protein/day/ha, if 500 carp of 1 kg is stocked. As the calculated minimum of protein requirement is 4.2 kg/ha, the available amount is more than 150% of the required one. Thus, we can be sure that the flesh of market-size carp will have a high quality, with complete, digestible protein and limited fat content.

**Figure 44:**
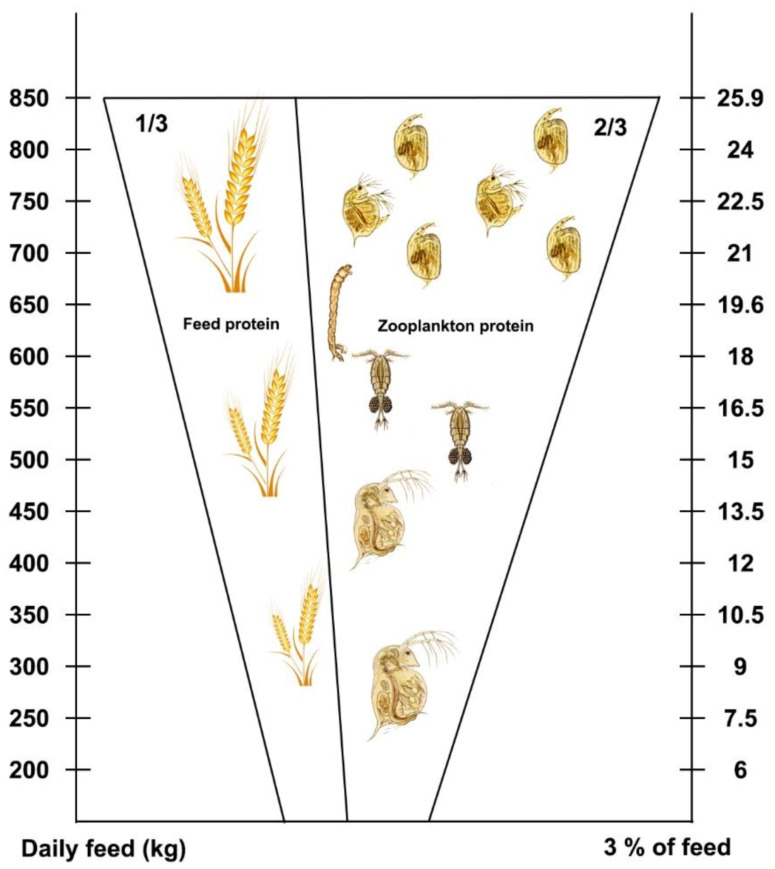
Protein requirement of market-size carp. A larger part of the protein originates from natural food full of essential components (such as amino and fatty acids). The growing stock needs an increased amount of grain, as well as higher proteins.

During the metabolically active season, carp are gradually growing; therefore, feed amount should also increase (Table 2). Consequently, the absolute amount of protein needed is growing as well. The connection between the actual daily feed and the protein requirement is demonstrated in Figure 44.

To provide the required zooplankton and zoobenthos as a natural food of a pond ecosystem is much cheaper than to feed with supplementary grains, which makes pond fish production economically more profitable. The effect of this natural food for carp on the profitability of total pond production is analysed in the next section.

When the zooplankton level of a pond is low, external protein sources can help to reach the required level. After soaking, additional soybean and other legumin seeds can be used for this purpose.

If farmers apply this advanced feeding technique based on protein requirements and zooplankton can be maintained at a relatively high level (more than 2 mL wet sediment of zooplankton per 100 L pond water), the daily feed of a carp stock can increase above 3% per day without reducing the quality of market-size carp. This is in contrast to ad libitum feeding, where overfeeding can cause a decline in meat quality (as fat level increases).

To keep the zooplankton amount at the required high level (over 2 mL/100 L water), the involvement of advanced agrotechnical methods (such as effective organic manuring, liming, recultivation of mud in the pond, etc.) is required, as presented in the previous sections.

### 3.3. Monitoring Growth during the Season

From spring stocking to autumn fishing, controlling and monitoring the growth and feed conversion ratios of fish are important tasks for the farmers. Every two to three weeks, control fishing needs to be completed. During the sampling activities, a representative amount of fish is collected. In large fishponds, it seems to be difficult to catch a high number of fish. The most efficient technique is to use throw nets on the feeding area, following the daily feeding.

During fish sampling, the weight of the collected fish is measured, and an average weight is calculated. This value is then compared with previous data to obtain the weight gain of the elapsed period. Farmers can estimate the volume of the population living in a pond, and by knowing the amount of feed transported into the pond between the two sampling periods, they can also count the food conversion ratio, namely how much food resulted in one kg of fish weight gained in that specific period. The average food conversion ratio in pond cultures is about 3.5—4.5 kg of feed/kg of fish weight.

Regular stock sampling provides a good opportunity to control fish health as well. If some signs of parasites or infections are detected, veterinary experts can provide advice on treatments.

### 3.4. Autumn Fishing

At the end of the active production season, when the temperature of pond water drops below 8–12 °C, the feeding activity of carp significantly drops as well. This is the time to start autumn fishing.

**Figure 45:**
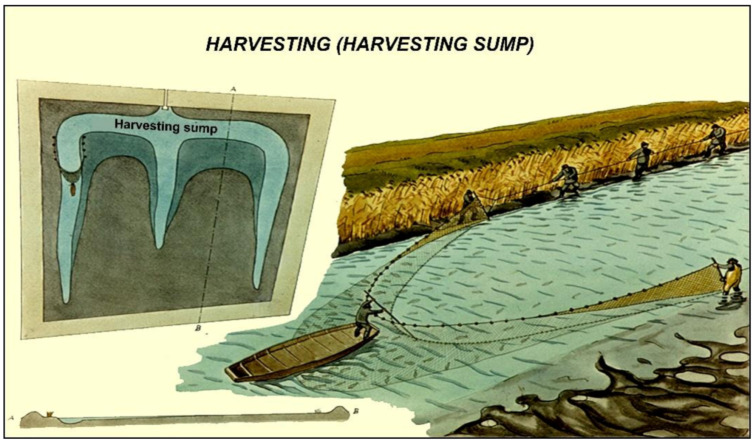
From the channel-shaped fish bed, the fish stock is removed by large nets.

**Figure 46:**
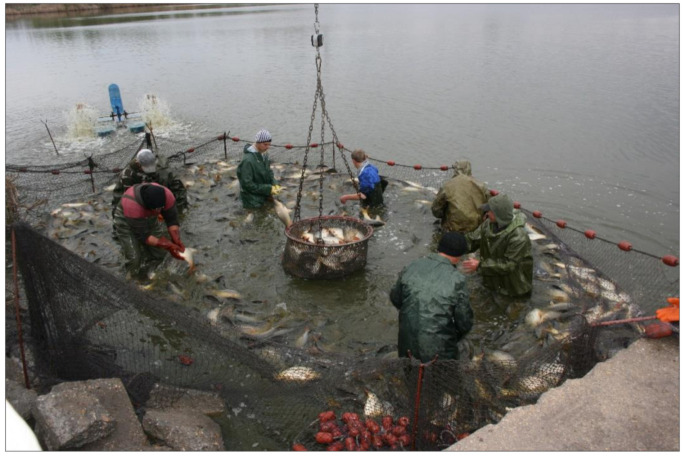
Fish with high oxygen needs, such as pikeperch, are the first ones to be collected by hand. Parallelly, extra oxygen is released by pumps or oxygen generators (Photo: A. Hegyi).

**Figure 47:**
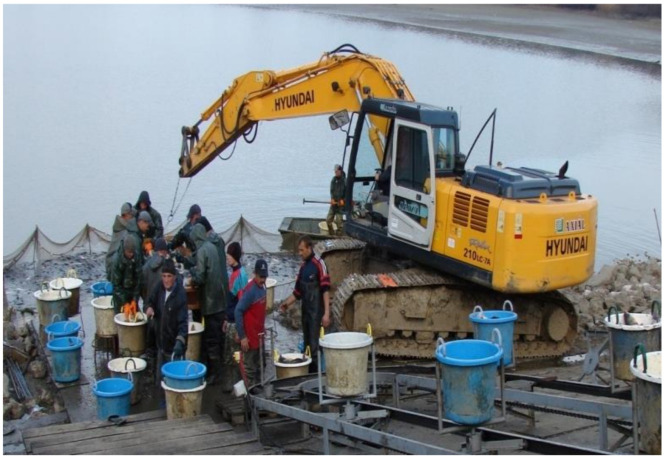
For movement, selection and transportation of mixed fish populations from large fishponds or water reservoirs, special machines are used due to their higher efficiency and the limited amount of labour.

To prepare for this activity, first the pond needs to be drained slowly, step by step. When this is finished, all fish are accumulated in the deeper parts of the pond (inside the fish bed), making it easier to catch fish in high densities within a limited area. After the fish are removed from the water, selection by size and species is the next step to perform (Figure 45, Figure 46 and Figure 47).

If wintering ponds are available in sufficient numbers, selected groups of fish are transferred to separate small ponds.

### 3.5. Wintering of Selected Fish Stocks in Small Fishponds

**Figure 48:**
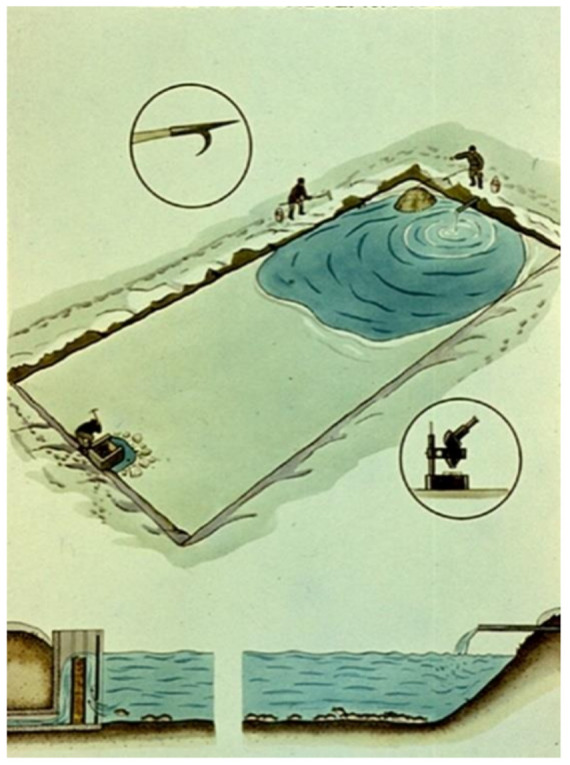
Fish spend the winter in small (1000 m^2^), deep ponds with good water supply, where the stocks live in high densities, with low metabolic activity (5–600 L/min water flow provides enough oxygen even for 5–10 tonnes of fish).

**Figure 49:**
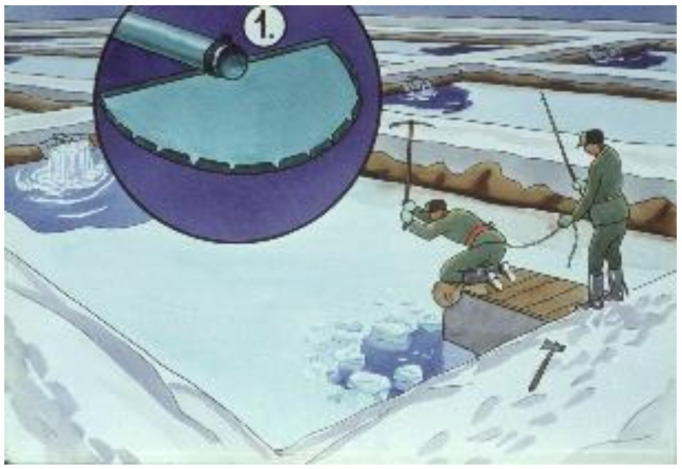
During wintering, monks are regularly cleaned from ice as regulated by rules of labour protection. A plate is placed under the inflow, which expands the affected water surface and thus increases oxygen solubility (1.: inlet pipe).

**Figure 50:**
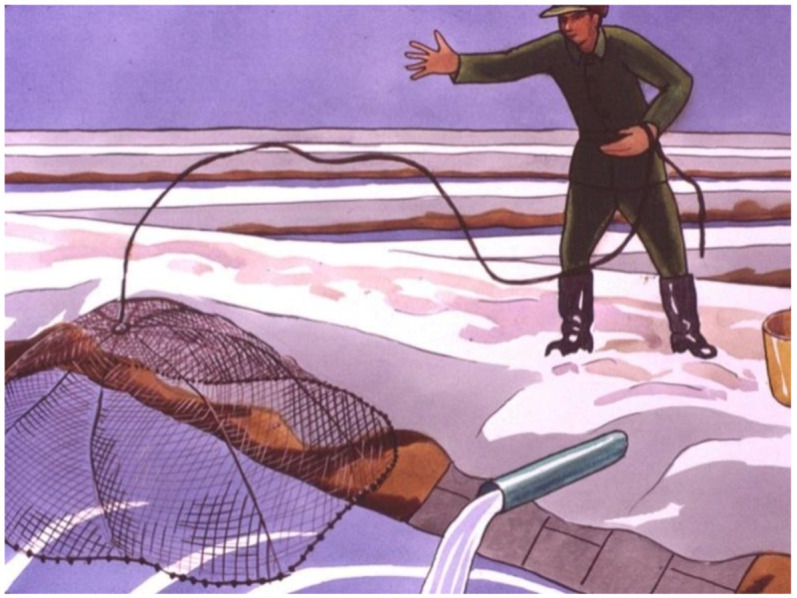
Fish samples taken for veterinary inspection by throw net. Accumulation of fish under the water inflow may indicate a parasitic or bacterial infection, which needs a veterinary inspection. Only live samples are suitable for microscopic investigations.

Wintering of fish is a quiet breeding period. The main task of fish farmers is to take care of the fish and regularly control their health (Figure 48, Figure 49 and Figure 50). In cold water, no fish feeding happens.

During springtime, when the water temperature starts to increase, after weighing, fish stocks from wintering ponds are transported to production ponds. With the stocking process, the vegetation season starts again (see Figure 40).

## 4. Evaluation of a Breeding Season Based on Food Conversion: The Ratio of Natural Production Built on Zooplankton

After the end of autumn, fishing farmers should analyse their seasonal production.

At first, total growth is determined as the difference between the initial and final weight per 1 ha. Some categories can be used:

(a) Total amount of fish after autumn fishing:
  ∑ All fish (kg) = N × w
  N = number of fish
  w = total weight of fish stock
  or:
(b) Total amount of fish can be calculated in a different way as well:

∑ All fish after autumn fishing (kg) == ∑ Fish weight stocked at spring + P_net_(kg)

where P_net_(kg) = the fish production of the whole season in kg.

To calculate the production for 1 ha, the total amount of produced fish should be divided by the size of the pond (ha).

(c) Net fish production can be divided into subunits:
  P_net_ = P_feed origin_ + P_natural food origin_

summer of food artificially released to one ha (kg)

$$P_{\mathrm{feed}\mathrm{origin}}=\frac{\mathrm{Total}\mathrm{amount}\mathrm{of}\mathrm{artificial}\mathrm{feed}\mathrm{released}\mathrm{to}1\mathrm{ha}}{3.5-4.5}$$

The amount of 3.5–4.5 is a practical value. In general, from this amount of grain feeds, 1 kg of fish is produced, if enough zooplankton is present in the water. The presence of zooplankton as natural food is a crucial condition!

P_calculated natural production_ = P _net production_ − P_production of feed origin_

The feed origin part of fish production comes from consuming energy-rich grains, while another important part derives from the consumption of renewable foods of natural origin, such as zooplankton and zoobenthos.

The main task of the fish farmer is to increase the fish production part of natural origin, as this is very cheap and generates high-quality fish flesh.

## 5. Environmental Effects and Carbon Footprint of Pond Fish Farming

All over the world, human foods of animal origin cause relatively high carbon footprints as production methods have high carbon dioxide and methane emissions. Culturing different plant products, such as grains, also increases the amount of carbon dioxide in the atmosphere due to the high fossil energy requirements of culturing procedures, among others.

In the case of carp production in ponds, the carbon footprint is not generated from the production of fish flesh of biological origin, as shown in Section 1. regarding carbon and energy circulation within water ecosystems, but rather, due to diffusion, the whole process fixes a certain amount of carbon dioxide from the atmosphere.

As analysed in Section 1. regarding the molecular level of inorganic carbon migration, when carbon dioxide enters or appears in pond water, it is fixed as calcium or magnesium hydrocarbon, keeping it in strong chemical bounds. Only water algae can absorb it through osmotrophy, so it is not possible for carbon dioxide to move from pond water into the atmosphere.

The efficient bioturbation of sedimented organic mud by carp within a water ecosystem result in a quick degradation followed by a rebuilding of organic compounds, thus modelling circular industry within aquaculture production, where no dangerous carbon emissions can be detected.

In pond fish production, only supplementary feeding can cause a carbon footprint when grains are cultured, as explained above.

Thus, as is justified in the fish production analysis section, a significant part of the total fish production is based on biological resources. Subsequently, pond carp production processes are basically among the rare, environmentally safe animal production methods.

## 6. Conclusions

The traditional pond carp breeding method in Central Europe introduced above is able to produce valuable fish meat in an economical way, as it utilizes free, renewable natural resources of fishponds. At the same time, it is environmentally friendly, since carbon dioxide produced or introduced into the system is fixed by strong covalent or ionic chemical bonds to calcium or magnesium cations, so fishponds also serve as carbon dioxide traps. The special food-seeking property of carp (bioturbation) continuously resuspends mud. As a result, the organic substance of algal origin generated during biological production does not accumulate in the sediment but is able to reach carp biomass through the food chain.

For that reason, the continuous reconstruction of carp-producing fishponds in operation for decades/centuries and the construction of new ones are definitely justified. It is expected that due to the pronounced advantages, carp farming will increase in the future.

Chinese carp production accounts for the the majority of world carp production. China produced >90% of the world’s carp fish [27]. Grass-carp-dominated ’Asian carp’ production, namely bighead carp, grass carp, silver carp and black carp (in 2020, total production was 13 million tons [28]), is colourful depending on the different production systems and environmental conditions of the huge country. The structure of the Chinese model differs from the European, carp-dominated system; therefore, comparing them is difficult. Everyone accepts that the Chinese polyculture is the most productive one in the world. Our manuscript focuses on the description of the carp breeding features of the Carpathian basin only. A comparative study of the two systems detailing data collection is needed, by using multivariate statistical analyses taking into account different economic, production and sociological contexts.

## Figures and Tables

**Table 1:** Data of stocking density and yield in a three-year-long carp production period.

	Stocking		Survival		Yield
	pcs/ha	kg/ha	pcs/ha	%	kg/ha
Fry—advanced fry	1–4 million	0	300,000–2 million	30–60	90–400
Fry—one summer old	300,000–600,000	0	25,000–70,000	5–30	400–1000
Advanced fry—one summer old	60,000–120,000	20–30	35,000–60,000	50–70	900–1400
One summer old—Two summers old	10,000–15,000	100–300	6000–10,000	50–70	1200–1800
Two summers old—market size	1000–2500	200–500	800–2000	60–80	1200–1600

**Table 2:** Percentage of the total weight of the fish stock. By the time the warm summer comes, this ratio can be increased up to 3% of the current weight of a stock. To determine the total weight of a fish population, regular samplings need to be completed every 2–3 weeks during the summer.

% of Carp Feed Delivered per Month												
Age Groups of Fish	March	April	May	June	July	August	September	October	November	December	January	February
One summer old	2	4	5	10	15	35	25	-	1	1	1	1
Two summers old	1	4	10	15	20	30	20	-	-	-	-	-
Three summers old	1	4	10	15	20	28	22	-	-	-	-	-

## Data Availability

Not applicable.
